# Genotypic and phenotypic characterization of multidrug resistant *Salmonella* Typhimurium and *Salmonella* Kentucky strains recovered from chicken carcasses

**DOI:** 10.1371/journal.pone.0176938

**Published:** 2017-05-08

**Authors:** Rizwana Tasmin, Nur A. Hasan, Christopher J. Grim, Ar’Quette Grant, Seon Young Choi, M. Samiul Alam, Rebecca Bell, Christopher Cavanaugh, Kannan V. Balan, Uma S. Babu, Salina Parveen

**Affiliations:** 1 Agriculture, Food and Resource Sciences, University of Maryland, Eastern Shore, Princess Anne, Maryland, United States of America; 2 University of Maryland Institute for Advanced Computer Studies, University of Maryland, College Park, Maryland, United States of America; 3 CosmosID Inc., Rockville, Maryland, United States of America; 4 Office of Applied Research and Safety Assessment, Center for Food Safety and Applied Nutrition, US Food and Drug Administration, Laurel, Maryland, United States of America; 5 Office of Regulatory Science, Center for Food Safety and Applied Nutrition, US Food and Drug Administration, College Park, Maryland, United States of America; Institut National de la Recherche Agronomique, FRANCE

## Abstract

*Salmonella* Typhimurium is the leading cause of human non-typhoidal gastroenteritis in the US. *S*. Kentucky is one the most commonly recovered serovars from commercially processed poultry carcasses. This study compared the genotypic and phenotypic properties of two *Salmonella enterica* strains Typhimurium (ST221_31B) and Kentucky (SK222_32B) recovered from commercially processed chicken carcasses using whole genome sequencing, phenotype characterizations and an intracellular killing assay. Illumina MiSeq platform was used for sequencing of two *Salmonella* genomes. Phylogenetic analysis employing homologous alignment of a 1,185 non-duplicated protein-coding gene in the *Salmonella* core genome demonstrated fully resolved bifurcating patterns with varying levels of diversity that separated ST221_31B and SK222_32B genomes into distinct monophyletic serovar clades. Single nucleotide polymorphism (SNP) analysis identified 2,432 (ST19) SNPs within 13 Typhimurium genomes including ST221_31B representing Sequence Type ST19 and 650 (ST152) SNPs were detected within 13 Kentucky genomes including SK222_32B representing Sequence Type ST152. In addition to serovar-specific conserved coding sequences, the genomes of ST221_31B and SK222_32B harbor several genomic regions with significant genetic differences. These included phage and phage-like elements, carbon utilization or transport operons, fimbriae operons, putative membrane associated protein-encoding genes, antibiotic resistance genes, siderophore operons, and numerous hypothetical protein-encoding genes. Phenotype microarray results demonstrated that ST221_31B is capable of utilizing certain carbon compounds more efficiently as compared to SK222_3B; namely, 1,2-propanediol, M-inositol, L-threonine, α-D-lactose, D-tagatose, adonitol, formic acid, acetoacetic acid, and L-tartaric acid. ST221_31B survived for 48 h in macrophages, while SK222_32B was mostly eliminated. Further, a 3-fold growth of ST221_31B was observed at 24 hours post-infection in chicken granulosa cells while SK222_32B was unable to replicate in these cells. These results suggest that *Salmonella* Typhimurium can survive host defenses better and could be more invasive than *Salmonella* Kentucky and provide some insights into the genomic determinants responsible for these differences.

## Introduction

Salmonellosis, caused by the Gram-negative bacterial genera, *Salmonella*, is one of the major causes of bacterial food-borne illness globally. Each year, an estimated 93.8 million cases of salmonellosis occurs worldwide [1]. In the United States, salmonellosis is the second leading cause of food-borne illnesses in humans [2], accounting for approximately 1 million cases, 19,336 hospitalizations and 378 deaths each year [2]. About 31% of all food-borne illness related deaths each year in the US are attributed to *Salmonella* infections [3] and the annual medical cost for salmonellosis in the US is estimated at $3.7 billion [4].

*Salmonella enterica*, belonging to the family, Enterobacteriaceae, consists of more than 2,500 serovars. *Salmonella* Enteritidis and *S*. Typhimurium are the two most common serovars that cause food-borne illnesses in the US [2]. However, *S*. Kentucky is one of the most frequently isolated serovars from poultry carcasses (ST152) in the US [5] and is sometimes attributed with one or more antibiotic resistant phenotypes [6–8]. Historically, *S*. Kentucky has rarely been associated with human illness; however, recently *S*. Kentucky (ST198) has been found to befrequently associated with human clinical cases in travelers to North Africa and South Asia [9–10]. Moreover, the global emergence of multidrug resistant *Salmonella* strains poses a serious threat to public health and has drawn significant interest to poultry industries, particularly in the poultry processing steps to control the level of contamination and transmission [11].

Among the various strategies to control the growth of *Salmonella* and other pathogenic microorganisms, chlorine in the form of sodium hypochlorite (HOCl), is used as a safe antimicrobial agent in commercial poultry processing plants during the immersion-chilling step. Sodium hypochlorite at a lower concentration (20 to 50 ppm) can kill *Salmonella* and other pathogens [12]. In addition, commercial poultry industries also used to treat these birds with various broad-spectrum antibiotics to prevent subclinical infections and to reduce the overall bacterial load in these birds [13]. Due to the growing concern of emerging resistant organisms, final guidelines were issued by the U.S. Food and Drug Administration (2013) to phase out the use of medically important antibiotics in livestock for production purposes [14]. Although antibiotic use is being phased out, current practice still allows for the use of broad-spectrum antibiotics in broiler flocks.

Recent reports on the isolation of multidrug resistant (MDR) *S*. Typhimurium and *S*. Kentucky isolates from commercially processed chicken carcasses raises concerns regarding the management practices used in this industry [6, 15–16]. How these *Salmonella* isolates survive chilling and chlorine treatment is an unanswered question. Of further concern is that some of these recovered *Salmonella* isolates, regardless of which serovar they belonged to, displayed resistance to ceftiofur (51.7%), a commonly used chemotherapeutic for *Salmonella* infected patients [6, 17]. Taken together, one has to question whether the use of antibiotics in the poultry industry is being managed effectively and the efficacy of chlorine treatment in controlling multidrug resistant *Salmonella* during processing.

In a previous study [6], our laboratory conducted research on the prevalence and antimicrobial resistance of *Salmonella* spp. isolated from processed poultry. Whole broiler carcasses were obtained directly from the downstream processing line at two selected points (pre- and post-chill) from a commercial processing plant. A significant number of recovered *Salmonella* isolates (45.8%) were resistant to an average of five or more antibiotics [6], and these isolates were further analyzed by PCR assays for selected virulence markers [16]. However, information is limited on the genotypic, phenotypic and immunologic properties of MDR *S*. Typhimurium and *S*. Kentucky strains surviving the immersion-chilling step in a commercial poultry processing plant. Therefore, this study was undertaken to identify similarities and differences between representative MDR *S*. Typhimurium and MDR *S*. Kentucky strains, using whole genome sequencing and comparative genomic analyses, phenotypic microarray, and macrophage killing and granulosa cell invasion. Characterization and comparison of these traits should lead to a better understanding of the processes that drive the evolution of this species through the loss of genes or acquisition of mobile genetic elements.

## Materials and methods

### Selection of strains and extraction of DNA for whole genome sequencing

*Salmonella* strains used in this study were isolated in a previous study [6] from commercially processed whole broiler carcasses collected from a processing plant in the mid-Atlantic region. A total of 309 *S*. Typhimurium (*n* = 75) and *S*. Kentucky (*n* = 234) isolates were recovered by Parveen et al. [6] and 89.5% of *S*. Typhimurium and 84.2% of *S*. Kentucky isolates were multidrug resistant. Among these characterized *Salmonella* strains [6], one multidrug resistant (MDR), post-chill *S*. Typhimurium (ST221_31B) and one MDR, post-chill *S*. Kentucky (SK222_32B) strains were selected for this study as a representative strain from these two serovars, as most of the isolates were MDR and all isolates contained virulence genes *invA* and *pagC*. Both strains used in this study, recovered from the same chicken carcass, were resistant to six different antibiotics: tetracycline, ampicillin, amoxicillin, cefoxitin, ceftiofur, and sulfisoxazole [6, 16].

### DNA extraction

*Salmonella enterica* strains ST221_31B and SK222_32B were sub-cultured from frozen stocks onto Tryptic Soy Agar (TSA) agar plates amended with 5% sheep blood and incubated overnight at 37°C. Single isolated colonies were inoculated into Tryptic Soy Broth (TSB) and incubated overnight at 37°C, with shaking. Cell pellets were harvested by centrifugation at 6000×g for 5 min, and genomic DNA was extracted with the QIAcube automated sample preparation platform, using the QIAamp DNA mini protocol (Qiagen, Valencia, CA, USA).

### Sequencing of *Salmonella* genomes

Extracted genomic DNA was quantified using a Qubit 2.0 Flurometer (Life Technologies (ThermoFisher, Waltham, MA, USA), diluted, and prepared for sequencing using the Nextera XT DNA Library Prep Kit (Illumina, San Diego, CA, USA). Whole genome sequencing was performed on the MiSeq platform (Illumina, San Diego, CA, USA), utilizing 500 cycles of paired-end reads. Fastq datasets were trimmed and *de novo* assembled with CLC Genomics Workbench version 7.0 (CLC bio, Aarhus, Denmark). The draft genome size of *S*. Typhimurium ST221_31B is 4,989,491 bp, on 72 contigs, with a G+C% content of 52.1, while the draft genome size of *S*. Kentucky SK222_32B is 5,005,607 bp, on 55 contigs, with a G+C% content of 51.9. The genome coverage at each position for *S*. Typhimurium ST221_31B and *S*. Kentucky SK222_32B was, on average, 69 and 75%, respectively. The draft genome sequence assemblies were annotated using Rapid Annotation using Subsystem Technology (RAST) [18].

### Data availability and accession numbers

The Whole Genome Shotgun projects for *S*. Typhimurium ST221_31B and *S*. Kentucky SK222_32B have been deposited at DDBJ/ENA/GenBank under the accession JUIT01000000 and JUIU01000000, respectively. The versions described in this paper are versions JUIT01000000.1 and JUIU01000000.1. The genome sequencing reads for *S*. Typhimurium ST221_31B and *S*. Kentucky SK222_32B have been deposited at the NCBI SRA under the accession numbers of SRS775470 and SRS775521, respectively.

### Phylogenomics

A phylogenetic tree of the two newly sequenced *Salmonella* strains was constructed with 28 previously sequenced *S*. *enterica* strains to establish evolutionary relationships. Orthologous regions were identified by comparison based on similarity and the set of orthologous regions for each Open Reading Frame (ORF) of a reference genome was identified according to nucleotide similarity, and aligned using the CLUSTALW2 program. The resultant multiple alignments were concatenated to form genome scale alignments, and resultant alignments were used to construct the neighbor-joining tree [19]. Maximum likelihood [20] and maximum parsimony [21] trees were also constructed. Single nucleotide polymorphisms (SNPs) analysis of *S*. Typhimurium strains and *S*. Kentucky strains was determined using Parsnp core genome multi-aligner from the Harvest SNP calling package [22].

### Comparative genome analysis

Two methods for whole genome comparison were used to identify genomic similarities and differences between newly sequenced strains with previously sequenced *S*. *enterica* reference genomes including plasmids [23]. A sequence based genome-to-genome comparison was carried out using the bidirectional BLASTP strategy of the RAST SEED Viewer [24], version 2.0, to compare strains ST221_31B and SK222_32B with *S*. *arizonae* (IIIa) 62:z4,z23:− strain RKS2980 (GenBank Accession NC_010067.1), *S*. Newport SL254 (GenBank Accessions NC_011080.1, NC_011079.1, and NC_009140.1), *S*. Paratypi B SPB7 (GenBank Accession NC_010102.1), *S*. Agona SL483 (GenBank Accessions NC_011149.1 and NC_011148.1), *S*. Typhi CT18 (GenBank Accessions NC_003198.1, NC_003384.1 and NC_003385.1), *S*. Cholerasuis SC-B67 (GenBank Accessions NC_006905.1, NC_006856.1 and NC_006855.1), *S*. Dublin CT_02021853 (GenBank Accessions NC_011205.1 and NC_011204.1), *S*. Enteritidis P125109 (GenBank Accession NC_011294.1), *S*. Gallinarum 287/91 (GenBank Accession NC_011274.1), *S*. Heidelberg SL476 (GenBank Accessions NC_011083.1, NC_011082.1 and NC_011081.1), *S*. Kentucky CVM29188 (GenBank Accessions ABAK02000001.1, CP001122.1, CP001121.1 and CP001123.1), and *S*. Typhimurium LT2 (GenBank Accessions NC_003197.1 and NC_003277.1). A subset of this comparison is shown in “S2 and S3 Figs”. Due to the graphical output limitations of the SEED Viewer software, only 9 (RKS2980, SL254, SC-B67, CT_02021853, P125109, 287/91, SL476, CVM29188 and LT2) out of 12 genomes were used for the comparison shown in “S2 and S3 Figs”. Additionally, the comparative distribution of *Salmonella* virulence determinants was carried out by querying the genomes against a web-based virulence factor database (VFDB) (http://www.mgc.ac.cn/VFs/), and the genome assemblies were submitted to the PlasmidFinder tool from CGE to elucidate plasmid incompatibility classes [25].

### Phenotype characterizations by Biolog phenotype microarray (PM)

Phenotypic microarrays (PM) of both strains were prepared according to the manufacturer’s specifications (Biolog, Inc., Hayward, CA). *Salmonella* Typhimurium (ST221_31B) and *S*. Kentucky (SK222_32B) were incubated overnight at 37°C on tryptic soy agar (TSA) plates. Sterile swabs were used to aseptically transfer cells from the TSA plates to tubes containing 16 ml of inoculation fluid-0 (IF-0) until a turbidity transmittance (T) of 42% ±1 was reached on the Biolog turbidity meter. A 1:5 dilution of the 42% T into IF-0+dye was performed to give a final cell density of 85% T. PM assays consisted of 10 panels (PM1, PM2A, PM3B, PM4A, PM5, PM6, PM7, PM8, PM9, PM10) which tested the strains’ ability to utilize different carbon, nitrogen, phosphorus, sulfur, and peptide nitrogen sources, as well as varying osmolytes, nutrient supplements and pH conditions. Antibiotic susceptibility assays for PM were not done, since both strains were tested for their sensitivity to15 different antimicrobials using traditional antimicrobial sensitivity testing [6]. After the cell suspensions were added to each PM plate, they were incubated for 48 hours in the OmniLog reader (Biolog). Bacterial growth, measured by the reduction of tetrazolium dye, was recorded every 15 minutes by the OmniLog charge coupled device analyzing camera. PM data analysis was performed using OmniLog® Phenotype™ MicroArray Software Release 1.2.

### Infection of cultured macrophages and assessment of intracellular growth of bacteria

*Salmonella* Typhimurium (ST221_31B) and *S*. Kentucky (SK222_32B) were grown overnight at 37°C in BHI (Brian heart infusion) broth in static culture and then washed twice with PBS (phosphate buffered saline, pH 7.4). The optical density (at 600 nm) was measured to adjust the bacterial cell density to 10^8^ colony forming units/ml (CFU/ml) and the bacterial suspension was further diluted in DMEM (Dulbecco’s Modified Eagle Medium). The bacterial suspension was added to 12-well plates at a multiplicity of infection (MOI) of 10 for both HD-11 and RAW 264.7 cells (ATCC TIB-71, Manassas, VA). HD-11 cells are a macrophage-like immortalized cell line derived from chicken bone marrow and transformed with the avian myelocytomatosis type MC29 virus [26]. Plates were centrifuged for 5 min to synchronize the infection followed by incubation at 37°C under 5% CO_2_ for 90 min for both macrophage cell lines. Subsequently, plates were washed three times with pre-warmed PBS (pH 7.4) and were incubated with media containing 100 μg/ml gentamicin for 1 h, followed by culture with DMEM containing 50 μg/ml gentamicin for the rest of the experiment. Intracellular killing of bacteria by macrophages was assessed by means of the standard gentamicin assay [27–28]. At the end of the infection period, the infected macrophages were treated with 100 μg/ml gentamicin to kill the extracellular bacteria. At 0 h, 24 h and 48 h of incubation after the start of infection, the supernatant was removed and cells were washed three times with DMEM and lysed with 0.1% Triton X-100. Serial dilutions of the lysates were plated onto BHI agar to allow colony formation for quantification of intracellular bacteria.

### Invasion of chicken ovarian granulosa cells and assessment of intracellular growth of bacteria

The assay procedure used for invasion of granulosa cells by *Salmonella* strains was as previously described [29] with minor modifications. Although ST221_31B and SK222_32B are isolates from broiler carcasses, *S*. Typhimurium is known to colonize poultry and contaminate eggs, and as such there are no egg related outbreaks associated with *S*. Kentucky. Briefly, actively laying hens were euthanized by cervical dislocation, and pre-ovulatory follicles were excised from the ovarian tissue. All animal procedures described herein were reviewed and approved by the Center for Food Safety and Applied Nutrition (CFSAN) committee for Institutional Animal Care and Use (IACUC). The granulosa layer was separated from the thecal layer as previously described [30] and dissociated in Type IV collagenase (1.5 mg/ml, Worthington Biochemical Corp., Lakewood, NJ, USA) containing M199 medium (Life Technologies, Grand Island, NY, USA). The viable dispersed cells were enumerated by trypan blue exclusion method and suspended in M199 medium supplemented with Bovine Serum Albumin (0.2%), Glucose (0.2%), Chicken Serum (4%), Fetal Bovine serum (4%) and Antibiotic-Antimycotic (1X) solutions (Life Technologies). Approximately 2×10^6^ granulosa cells were seeded onto 60 mm cell culture dishes (Corning, Tewksbury, MA, USA), and grown to form a monolayer for 48 h at 37°C with 5% CO_2_. Logarithmic cultures of *Salmonella* strains (ST221_31B and SK222_32B) grown in aerated buffered peptone water at 37°C were normalized at OD 600 nm to 2×10^8^ CFU/ml. The ovarian granulosa cells (2×10^6^) in antibiotic-free medium were infected with *Salmonella* strains at a MOI of 30. The cells were incubated for 1.5 h at 37°C and rinsed with gentamicin containing medium (50 μg/ml, Life Technologies). Non-internalized bacteria were removed by washing and killed by gentamicin treatment. After 1 h, cells were incubated at a lower gentamicin concentration (25 μg/ml) for a total of either 4 h or 24 h post infection. After infection, granulosa cells were again enumerated, washed with gentamicin-free medium and lysed with 0.2% Triton X-100. The intracellular bacteria were collected and enumerated on Xylose Lysine Desoxycholate agar using an Eddy Jet 2 spiral plater and flash & go colony counter (Neutec Group, Inc. Farmingdale, NY, USA). *Salmonella* counts were expressed as colony forming units per granulosa cells lysed (CFU/granulosa cell).

Colony forming unit count data are from one representative experiment done at least 4 times. Percent survival data were pooled from 3–4 experiments. Data were mean ± SEM from representative experiment (*p* < 0.05 by *t*-test, unpaired).

## Results

### Phylogenomics of *S*. Typhimurium (ST221_31B) and *S*. Kentucky (SK222_32B)

**Fig 1:**
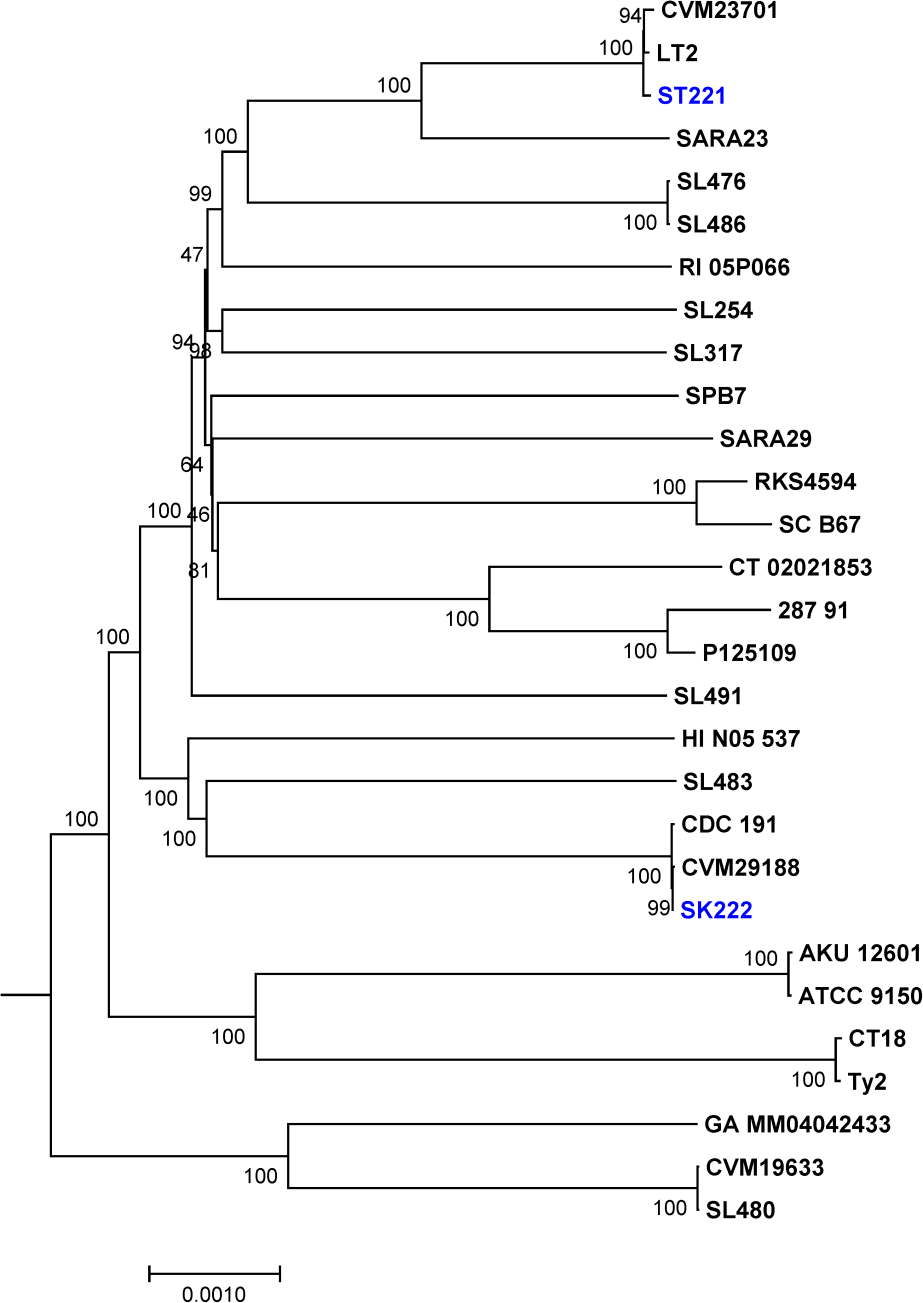
The rooted phylogenetic tree based on core genome of *S*. *enterica*. Neighbor-joining tree was constructed based on homologous alignment of 1,185 conserved ORFs (1,113,790 bp). The tree was rooted with *Salmonella enterica* subsp. arizonae serovar 62:z4,z23:- str. RSK2980. Bar represents 0.001 substitutions per site.

The genomes of the two newly sequenced strains ST221_31B (ST19 [Sequence Type 19], EBG1 [eBURST group 1]) and SK222_32B (ST152, EBG15) were compared with 28 previously sequenced, publicly available *Salmonella* genomes (“Table 1”) and a phylogenetic tree was constructed. The phylogeny of all 30 *S*. *enterica* strains were inferred by constructing a genetic relatedness neighbor-joining tree using homologous alignment of orthologous 1,185 highly conserved protein coding genes, representing approximately 1.1 million base-pairs (~1,113,790 bp) of a *Salmonella* core genome. The tree was rooted with *Salmonella enterica* subsp. arizonae serovar 62:z4,z23:-str. RSK2980 (“Fig 1”). The rooted *Salmonella* tree demonstrated fully resolved bifurcating patterns with varying levels of diversity and placed both *S*. Kentucky and *S*. Typhimurium strains into distinct monophyletic clades, suggesting that these two *S*. *enterica* serovars evolved into distinct evolutionary lineages from their most common phylogenetic ancestor. The newly sequenced Typhimurium strain (ST221_31B) branched with other Typhimurium genomes, LT2 and CVM23701, with *S*. *enterica* serovar Saintpaul (SARA23) being the closest phylogenetic out-group. This observation indicated that serovar Typhimurium shared a common ancestry with serovar Saintpaul before these two serovars evolved independently. Similarly, the newly sequenced Kentucky strain (SK222_32B) formed a tight monophyletic clade with two other Kentucky genomes, CDC191 and CVM29188 (“Fig 1”). *S*. *enterica* serovars Agona (SL483) and Weltevreden (HI_N05-537) appeared as out-groups indicating their shared ancestry with serovar Kentucky. Maximum likelihood and maximum parsimony trees are shown in “S1(A) and S1(B) Fig” respectively.

**Table 1:** List of strains used for phylogenetic analysis of *S*. *enterica* genomes.

Strain	Serovar	Source Type	Accession no.	Sequence type
ST221_31B*	**Typhimurium**	**Processed whole chicken carcass**	**JUIT01000000**	**ST-19**
LT2	Typhimurium	Human	AE006468	ST-19
CVM23701 (SL474)	I 4,_5_,12:i:_	Human	ABAO00000000	ST-19
SK222_32B*	**Kentucky**	**Processed whole chicken carcass**	**JUIU01000000**	**ST-152**
CDC 191 (SL479)	Kentucky	Human	ABEI00000000	ST-152
CVM29188 (SL475)	Kentucky	Chicken breast	ABAK00000000	ST-152
SL483	Agona	Human	CP001138	ST-13
SL477	Dublin	Human	CP001144	ST-10
RI_05P066 (SL485)	Hadar	Human	ABFG00000000	ST-33
SL476	Heidelberg	Ground turkey	CP001120	ST-15
SL486	Heidelberg	Human	ABEL00000000	ST-15
SL474	I 4,_5_,12:i:_	Human	ABAO00000000	ST-19
SL478	Javiana	Human	ABEH00000000	ST-24
SL254	Newport	Human	CP001113	ST-45
SL317	Newport	Human	ABEW00000000	ST-5
SARA23	Saintpaul	Human	ABAM00000000	ST-50
SARA29	Saintpaul	Human	ABAN00000000	ST-95
CVM19633 (SL473)	Schwarzengrund	Dehydrated chili	CP001127	ST-322
SL480	Schwarzengrund	Human	ABEJ00000000	ST-322
SL491	Virchow	Human	ABFH00000000	ST-16
HI_N05-537 (SL484)	Weltevreden	Scallops	ABFF00000000	ST-365
RKS2980	62:z4,z23:	NA	CP000880	NA
SC-B67	Choleraesuis	Human	AE017220	ST-66
P125109	Enteritidis	Human	AM933172	ST-11
287/91	Gallinarum	Chicken	AM933173	ST-331
AKU_12601	Paratyphi A	Human	CP000026	ST-85
ATCC 9150	Paratyphi A	Laboratory strain	CP000026	ST-85
SPB7	Paratyphi B	Human	CP000886	ST-307
RKS4594	Paratyphi C	Human	CP000857	ST-114
CT18	Typhi	Human	AE014613	ST-2
Ty2	Typhi	NA	AE014613	ST-1

*Strains sequenced in this study; NA, not available.

**Fig 2:**
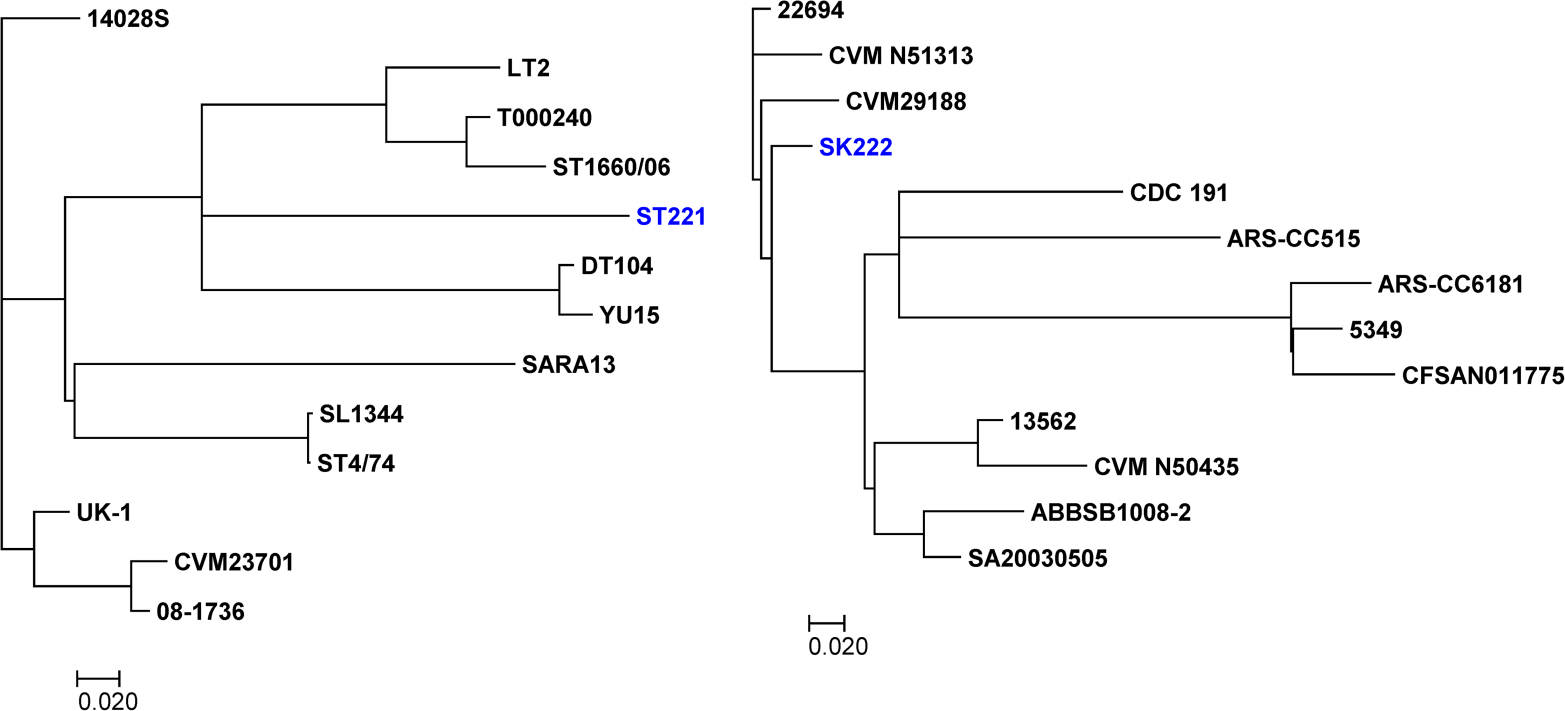
SNP-based phylogenetic tree. (A) *Salmonella enterica* serovar Typhimurium (ST221_31B) and (B) *Salmonella enterica* serovar Kentucky (SK222_32B) constructed using 2,432 (ST19) and 650 (ST152) SNPs identified within each group, respectively.

To elucidate genomic differences between genomes within each serovar cluster, entire genomes were compared for high resolution SNPs analysis. 2,432 (ST19) SNPs were identified among the *S*. Typhimurium ST221_31B, 14028 S, LT2, T000240, ST1660/06, DT104, YU15, SARA13, SL1344, ST4/74, UK-1, CVM23701 and 08–1736 genomes (“Fig 2A”), whereas 650 (ST152) SNPs were found among the *S*. Kentucky SK222_32B, 22694, CVMN51313, CVM29188, CDC191, ARS-CC515, ARS-CC6181, 5349, CFSAN011775, 13562, CVMN50435, ABBSB1008-2 and SA20030505 genomes (“Fig 2B”). In general, SNPs were distributed stochastically within Kentucky and Typhimurium genomes, without indication of mutational hotspots (data not shown).

### Comparative genomics of ST221_31B and SK222_32B

General features of the newly sequenced ST221_31B and SK222_32B genomes are given in “S1 Table”. Bi-directional BLAST approach was used to compare ST221_31B and SK222_32B genomes with nine previously sequenced *Salmonella* genomes (“S2 and S3 Figs”, respectively). In each figure (“S2 and S3 Figs”), colored arrows indicate examples of dissimilar gene content, such as insertion of mobile genetic elements (e.g., transposons, integrons) or plasmids in the reference genome, either ST221_31B, or SK222_32B, respectively, or deletion of genomic islands in one to all of the comparison genomes. In addition, a number of dissimilar smaller genomic islands, such as smaller prophage and prophage-like elements, characterized operons, and hypothetical proteins were also identified in both ST221_31B and SK222_32B strains. Detailed information is given in “S2 and S3 Tables”, respectively.

When the genomes of ST221_31B and SK222_32B were compared with a panel of twelve diverse *Salmonella* genomes, we identified 66 genomic regions that were present in both new genomes but missing from at least one comparison genome. Not surprisingly, the majority (59) of these dispensable genetic elements were found to be missing in the genome of *Salmonella enterica* subsp. *arizonae* strain RKS2980. These included the genetic determinants of phenotypic traits used to distinguish subsp. *enterica*, from this subspecies; namely, L(+)-tartrate and galactitol utilization, as well as operons for the utilization of several other putative carbon substrates, including allantoin, D-galacturonate and D-glucuronate, glycerate, L-ascorbate, L-idonate, glucosaminate, mannose, gentisate, L-rhamnonate, and xanthosine, *stfABCDEFG* and *stdABCD* π-fimbriae, *safABCD* γ_3_-fimbriae, *lpfABCDE* and *sthABCDE* γ_1_-fimbriae, and β-fimbriae clusters, and several membrane-associated and stress-response proteins. The genome of *Salmonella enterica* subsp. *enterica* Typhi CT18 was also missing several [29] of the genomic regions identified.

A total of 20 genomic regions were identified in the genome of ST221_31B, but absent in SK222_32B. These included three prophages, three prophage-like elements and two large integrons. We assume that most of the hypothetical or poorly annotated proteins encoded in these elements are phage-related proteins and toxin/antitoxin pairs; however, one of the phage-like elements encodes the O-antigen acetylase gene, *oafA*, responsible for the O5 epitope. For ST221_31B, there is a frameshift mutation in this gene, which results in loss of function, indicative of the Copenhagen variant of Typhimurium [31]. In addition, operons responsible for the utilization of D-tagatose, D-glucitol/D-sorbitol and L-sorbose, D-galactonate, and myo-inositol, and a fructose-like phosphotransferase system (PTS) were present in the genome of ST221_31B, as well as the cluster of genes that confer the ability to utilize sialic acid. Twenty two genomic regions were identified in the genome of SK222_32B, which were absent from the genome of ST221_31B. Among these, we identified one prophage, and five prophage-like elements. One of these elements harbors a four gene operon that is responsible for the DNA degradation phenotype, *dndBCDE*. The presence of this functional cluster results in strains that are non-typeable by pulsed-field gel electrophoresis (PFGE) due to degradation of the DNA. We also identified a transposon, which harbors a κ-fimbriae gene cluster (*pef*). In addition to these mobile genetic elements, we identified two additional fimbriae clusters, the *steABCDEF* π-fimbriae and the *staABCDEFD* γ4-fimbriae, and gene clusters encoding a “cryptic” mannitol transport system, the *ydj* unknown carbohydrate utilization system, and an arsenic resistance pump.

A total of five plasmids were found; two were found in the genome of ST221_31B and three in the genome of SK222_32B. A homologous Incl1 incompatibility class plasmid of approximately 100 kbp in size was found in the genomes of ST221_31B and SK222_32B. This plasmid was highly similar (99% identity, 99–100% query coverage) to plasmids from other *Salmonella* isolates collected from poultry; namely pN13-1290_98 (serovar Heidleburg, turkey meat, GenBank Accession CP012936.1), pCVM29188_101 (serovar Kentucky, poultry, GenBank Accession CP001121.1), pSA02DT1068701_99 (serovar Heidleburg, turkey meat, GenBank Accession CP012923.1), p12-4374_96 (serovar Heidleburg, turkey meat, GenBank Accession CP012929.1), with each plasmid displaying slight differences, apparently driven by transposases and insertion sequences (“S4B Fig”). Interestingly, this plasmid group is also highly similar to plasmid pSTM709 (GenBank Accession NC_023915), which harbors beta lactamase CMY-2, from a serovar Typhimurium isolate that is believed to be the first Amp-C producing *S*. Typhimurium strain in Uruguay. Phylogenetic reconstruction of the nucleotide sequence of this plasmid (“S4A Fig”) indicates a high degree of mobility, as its phylogeny is completely inconsistent with the *Salmonella* core genome phylogeny (“Fig 1”). This shared conjugative plasmid harbors the *bla*_CMY-2_ gene, conferring resistance to cephalosporins, the *blc* gene, and the *sugE* gene, which confers resistance to quaternary ammonium compounds, in a gene cassette flanked by mobility genes [32].

In addition to the shared Incl1 plasmid described above, strain ST221_31B harbors an IncA/C2 incompatibility class plasmid, which is estimated at 106,610 bp in size. It is contained on two contigs, ctgs. 2 and 56, which have a 20 bp overlap. It was found to be most similar, in terms of nucleotide identity, to an unnamed plasmid from the genome of *S*. Typhimurium strain CFSAN001921 (GenBank Accession CP006050.1), which was also isolated from chicken breast [33]. However, the plasmid from strain ST221_31B is an estimated 116,358 bp smaller in size than that of strain CFSAN001921, missing this segment as one large piece of DNA located on the 3’ end. Present on this plasmid are a *tetRA* gene cluster and a mercury resistance operon.

In addition to the shared Incl1 plasmid described above, strain SK222_32B harbors an IncFIB/IncFIIA (two origins of replication) incompatibility class plasmid, which is nearly identical to plasmid CVM29188_146 (GenBank Accession CP001122.1), and an IncX1 incompatibility class plasmid, which is nearly identical to plasmid pCVM29188_46 (GenBank Accession CP001123.1) [32]. Of interest, genes responsible for streptomycin and tetracycline resistance are encoded in the larger F-type conjugative plasmid, along with two siderophore gene clusters, aerobactin and salmonchelin, and the *sitABCD*-encoded manganese ACB transporter.

Virulence determinants found in both ST221_31B and SK222_32B are shown in “Table 2” (Sequence and locus tag of these virulence factors can be accessed at http://www.mgc.ac.cn/VFs).

**Table 2:** Virulence determinants of ST221_31B and SK222_32B, as determined by querying each genome against the virulence factors of pathogenic bacteria database.

Name	ST221_31B	SK222_32B	Gene	Functional Annotation
Vi antigen	-	-	*vexEDCBA/tviEDCBA*	Capsule
Agf/Csg	+	+	*csgGFEDBAC*	Fimbrial adherence determinants
Bcf	+	+	*bcfABCDEFG*	Fimbrial adherence determinants
Fim	+	+	*fimAICDHFZYW*	Fimbrial adherence determinants
Lpf	+	+	*lpfEDCBA*	Fimbrial adherence determinants
Peg	-	-	*pegDCBA*	Fimbrial adherence determinants
Saf	+	+	*safABCD*	Fimbrial adherence determinants
Sef	-	-	*sefABCD*	Fimbrial adherence determinants
Sta	-	-	*staGFEDCBA*	Fimbrial adherence determinants
Stb	+	+	*stbABCDE*	Fimbrial adherence determinants
Stc	+	+	*stcDCBA*	Fimbrial adherence determinants
Std	+	+	*stdCBA*	Fimbrial adherence determinants
Ste	-	+	*steABCDEF*	Fimbrial adherence determinants
Stf	+	+	*stfACDEFG*	Fimbrial adherence determinants
Stg	-	-	*stgABCD*	Fimbrial adherence determinants
Sth	+	+	*sthEDCBA*	Fimbrial adherence determinants
Sti	+	+	*stiABCH*	Fimbrial adherence determinants
Stj	+	+	*stjBC*	Fimbrial adherence determinants
Stk	-	+	*stkGFEDCBA*	Fimbrial adherence determinants
Tcf	-	-	*tcfABCD*	Fimbrial adherence determinants
Mig-14	+	+	*mig-14*	Macrophage inducible gene
Mg2+ transport	+	+	*mgtBC*	Magnesium uptake
MisL	+	+	*misL*	Non fimbrial adherence determinants
RatB	+	-	*ratB*	Non fimbrial adherence determinants
ShdA	+	+	*shdA*	Non fimbrial adherence determinants
TTSS (SPI-1 encode)	+	+	*SPI-1*	Secretion system
TTSS (SPI-2 encode)	+	+	*TTSS*	Secretion system
TTSS effectors translocated via both systems	+	+	*slrP*	Secretion system
TTSS-1 translocated effectors	+	+	*sopB*	Secretion system
TTSS-1 translocated effectors	+	+	*sopE2*	Secretion system
TTSS-1 translocated effectors	+	+	*spoA*	Secretion system
TTSS-1 translocated effectors	+	+	*avrA*	Secretion system
TTSS-1 translocated effectors	+	+	*sptP*	Secretion system
TTSS-1 translocated effectors	+	+	*sipA*	Secretion system
TTSS-1 translocated effectors	+	+	*sipC*	Secretion system
TTSS-1 translocated effectors	+	+	*sipB*	Secretion system
TTSS-1 translocated effectors	+	+	*sopD*	Secretion system
TTSS-2 translocated effectors	+	-	*ssel*	Secretion system
TTSS-2 translocated effectors	+	+	*pipB*	Secretion system
TTSS-2 translocated effectors	+	+	*sifA*	Secretion system
TTSS-2 translocated effectors	+	+	*ssaB*	Secretion system
TTSS-2 translocated effectors	+	+	*sseF*	Secretion system
TTSS-2 translocated effectors	+	+	*sseG*	Secretion system
TTSS-2 translocated effectors	+	+	*sifB*	Secretion system
TTSS-2 translocated effectors	+	+	*sseJ*	Secretion system
TTSS-2 translocated effectors	+	-	*sseK2*	Secretion system
TTSS-2 translocated effectors	+	-	*sspH2*	Secretion system
TTSS-2 translocated effectors	+	+	*sseL*	Secretion system
TTSS-2 translocated effectors	-	-	*gogB*	Secretion system
TTSS-2 translocated effectors	+	+	*pipB2*	Secretion system
TTSS-2 translocated effectors	+	+	*sseK1*	Secretion system
SodCl	+	-	*sodCl*	Stress protein
Typhoid toxin	-	-	*cdtB/pltAB*	Toxin
PhoPQ	+	+	*phoPQ*	Two component system

Both serovars harbored genes for fimbrial adhesion and type III secretion system (TSS3). However, *sseI*, *sseK2*, *sspH2*, and *sodCl* genes were only present in ST221_31B. Of interest, T3SS-2 effector, SspH2, is conserved among most *Salmonella* serovars and translocated through T3SS2 (Type III secretion system 2) [34]. And, *Salmonella* Typhimurium periplasmic superoxide dismutase SodCI is encoded by the Gifsy-2 prophage. This is required for protection of non-typhoidal bacteremia associated strains of *Salmonella* against phagocyte oxidative burst [35].

### Phenotype characterizations by phenotype microarray

Both *Salmonella* Typhimurium (ST221_31B) and *S*. Kentucky (SK222_32B) strains were characterized phenotypically using the OmniLog® PM system. Of the 960 conditions tested, only 22 variable biochemical traits were identified. ST221_31B was found to utilize 14 carbon sources that strain SK222_32B did not; namely, 1,2-propanediol, α-hydroxyglutaric acid-g-lactone, L-threonine, α-D-lactose, β-methyl-D-glucuronic acid, M-inositol, N-acetyl-D-mannosamine, D-tagatose, adonitol, formic acid, acetoacetic acid, L-tartaric acid, α-ketoglutaric acid, and D-galactonic acid-g-lactone as well as one nitrogen source, L-cysteine,. Conversely, strain SK222_32B was found to be more resistant to certain stress conditions, as compared to ST221_31B (“Table 3”).

**Table 3:** Phenotypic microarray substrate utilization.

Substrate	ST221_31B	SK222_32B
1,2-Propanediol	*	
α-Hydroxyglutaric Acid-γ-Lactone	*	
L-Threonine	*	
α-D-Lactose	*	
m-Inositol	*	
N-Acetyl-D-Mannosamine	*	
Adonitol	*	
Formic Acid	*	
Acetoacetic Acid	*	
α-Ketoglutaric Acid	*	
D-Galactonic Acid-γ-Lactone	*	
β-Methyl-D-Glucuronic Acid	*	
D-Tagatose	*	
L-Tartaric Acid	*	
L-Cysteine	*	
Ammonia		*
O-Phospho-L-Threonine		*
D,L-Ethionine		*
60mM Sodium Nitrate		*
80mM Sodium Nitrate		*
100mM Sodium Nitrate		*
pH 4.5	*	

*indicates significantly better utilization of the substrate based on area under the curve of the parametric data given by OnmiLog® PM software.

### Intracellular killing of *Salmonella* in macrophage

**Fig 3:**
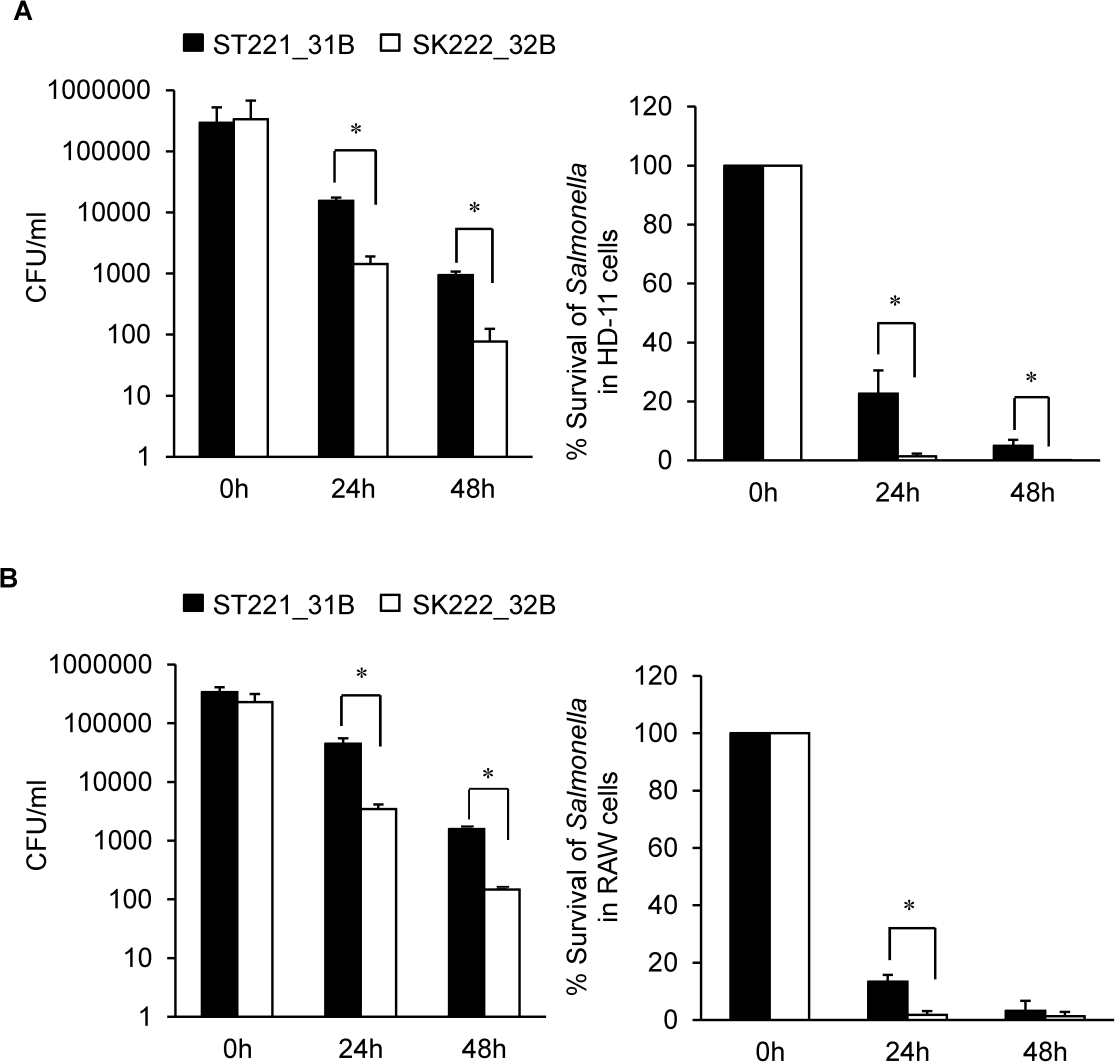
Intracellular survival of *Salmonella* strains in macrophages. ■ ST221_31B, □ SK222_32B. (A) HD-11 cells (chicken macrophages) or (B) RAW 264.7 (murine macrophages) were infected with *S*. Typhimurium (ST221_31B) and *S*. Kentucky (SK222_32B) at an MOI of 10. The number of intracellular bacteria (CFU/ml) was determined by plating serial dilutions of cell lysates on BHI agar (left panels). CFU count data are from one representative experiment done at least 4 times. Percent survival data (right panels) are pooled from 3–4 experiments. Data are mean ± SEM from representative experiment. *, p < 0.05 by t-test (unpaired).

To evaluate the intracellular survival of ST221_31B and SK222_32B in macrophages, HD-11 and RAW 264.7 cell cultures in a standard gentamicin protection assay or macrophage killing assay were used [27]. “Fig 3” shows the CFU counts of viable intracellular *Salmonella* recovered from macrophage lysates obtained at 24 h and 48 h after infection of macrophages. *Salmonella* Typhimurium (ST221_31B) survival was significantly higher compared to *S*. Kentucky (SK222_32B). By 48 h, SK222_32B was almost completely eliminated while ST221_31B was reduced to approximately 10% of the CFU at time 0 h.

### Invasion and growth of *Salmonella* in chicken ovarian granulosa cells

**Fig 4:**
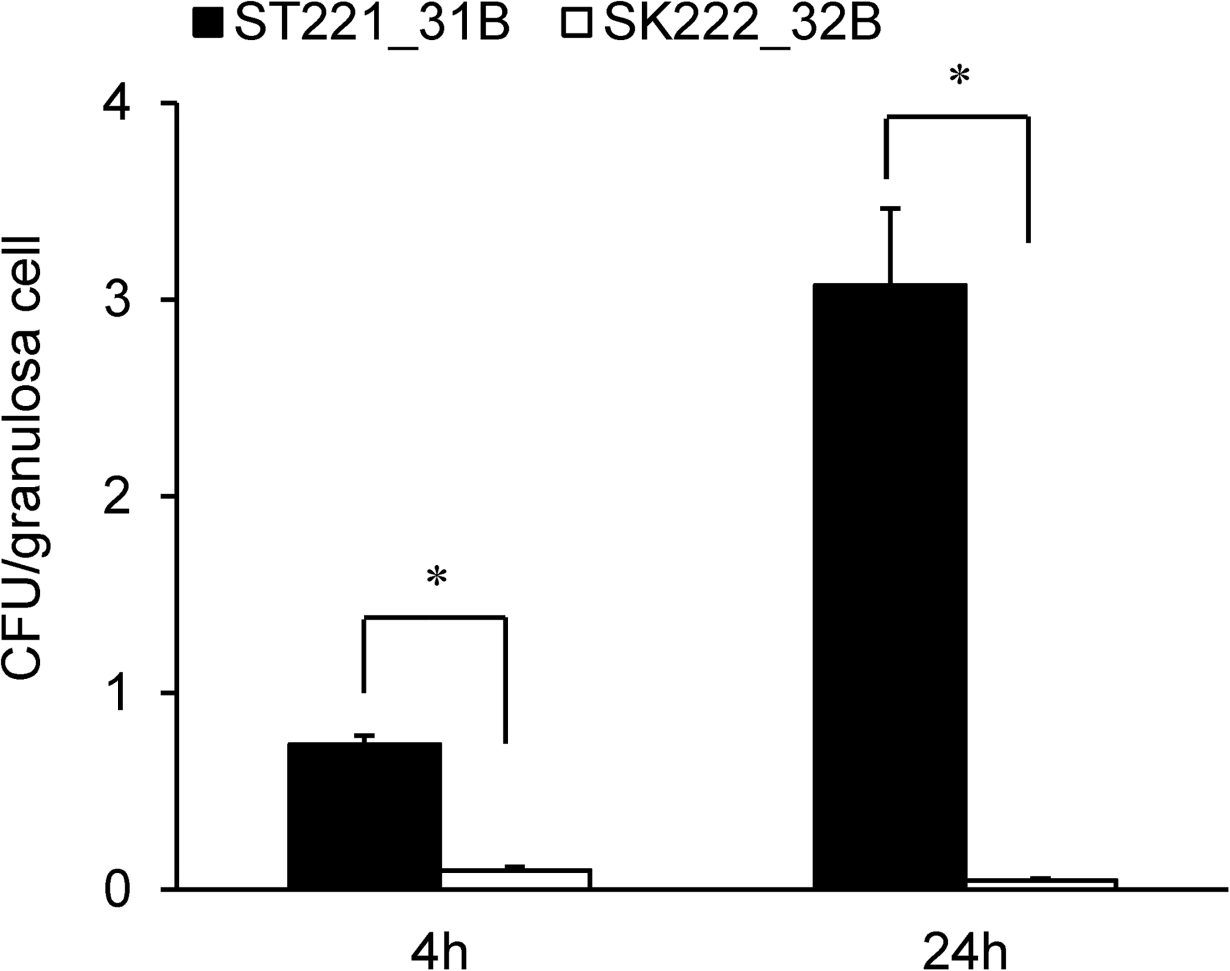
Intracellular survival of *Salmonella* strains in granulosa cells. ■ ST221_31B, □ SK222_32B. Ovarian granulosa cells were infected with *S*. Typhimurium (ST221_31B) and *S*. Kentucky (SK222_32B) at an MOI of 30. *, p < 0.05 by t-test (unpaired).

The invasiveness and replication potential of *S*. Typhimurium (ST221_31B) and *S*. Kentucky (SK222_32B) was evaluated in an *ex vivo* chicken ovarian granulosa cell model. As shown in “Fig 4”, SK222_32B was unable to replicate in the granulosa cells while, a 3-fold growth of ST221_31B was observed by 24 h post infection.

## Discussion

Despite extensive research efforts to characterize thousands of *Salmonella* strains every year by serotyping, pulsed-field gel electrophoresis (PFGE) fingerprinting, and antimicrobial resistance profiling by food safety stakeholders, food-borne *S*. *enterica* infection is still a major concern worldwide [36]. The genetic basis underlying the phenotypic diversity of *S*. *enterica* is not fully understood. However, some studies using comparative genomics have suggested that host specificity (e.g., humans, farm animals and birds) of *S*. *enterica* is accompanied driving factor in the loss or accumulation of genes or pseudogenes, resulting in the evolution of new lineages or sub-lineages from ancestral species [36]. Interestingly, these different sub-lineages of *S*. *enterica* can also differ by their antigenic presentation, virulence, and antimicrobial resistance phenotypes. Phylogenetic analysis based on phenotypic and genotypic characteristics suggest that the evolution of known *S*. *enterica* is caused by (i) the loss of coding sequences that directly affects the organism’s metabolic functions, and (ii) the acquisition of horizontally transferred phage and plasmid DNA, which confers virulence and resistance phenotypes leading to increased specialization of particular serovars, sub-types, or strains [36]. Therefore, a better understanding of each genotype is critical to predict its pathogenic potential, as well as to control overall disease burden.

Both of the newly sequenced strains (ST221_31B and SK222_32B) were attributed with MDR phenotypes [6]. Whole genome sequencing confirmed that both the strains possess multiple large conjugative plasmids conferring multidrug resistance phenotypes. An Incl1 plasmid, similar to pCVM29188_101 [32], harboring a bla_CMY-2_ gene was found in both strains, ST221_31B and SK222_32B [37], In addition to the IncI1 plasmid homologous to plasmid pCVM21988_101, the genome of strain SK222_32B also contains plasmids homologous to plasmid pCVM29188_146, which harbors genes responsible for streptomycin, tetracycline, and colicin resistance, and plasmid pCVM29188_46, which contains largely hypothetical or uncharacterized protein-encoding genes. Additionally, the genome of ST221_31B also contained an IncA/C2 class plasmid, which harbors genes for tetracycline resistance. Among the plasmids identified which carry resistance genes, it is interesting to note that several mobile element genes and transposonable elements are present on these plasmids, and often are flanking these resistance genes and gene cassettes. This arrangement can contribute to even greater mobility of antibiotic resistance gene determinants, as has been recently proposed among *Klebsiella pneumoniae* carbapenemase (KPC)-producing enteric isolates in clinical settings [38].

*Salmonella* Typhimurium LT2 contains a 94.7-kb virulence plasmid encoding the 7.8-kb *spv* operon that is associated with survival and growth in macrophages [39]. The exact role of the virulence plasmid in pathogenesis is not clear. However, evidence suggests that *spv* genes enable *S*. Typhimurium to infect the spleen and the liver by increasing the rate of bacterial replication within host cells. Plasmid-cured strains are able to colonize and persist in spleen and liver but their growth is controlled by host defense mechanisms [39]. Previous findings suggest that the virulence plasmids control the immune reaction, including complement activation (serum sensitivity), of the animal host in favor of the infecting *Salmonella* serovars [40]. The virulence plasmid has been frequently isolated and detected from clinical and field strains (mostly from infected animals and birds) of *S*. Typhimurium [41]. Interestingly, this virulence plasmid was not detected in *S*. Typhimurium ST221_31B. This result indicated that, all *S*. Typhimurium strains recovered from processed chicken carcasses may not harbor virulence plasmid.

The occurrence of serovar- and strain- specific conserved coding sequences, insertion and deletion of genomic islands and acquisition of mobile genomic elements and plasmids is a very common observation among *S*. *enterica* isolates [36]. Several genomic differences were detected in the genomes of both *S*. Typhimurium ST221_31B and *S*. Kentucky SK222_32B. Not surprisingly, these include plasmids and mobile genetic elements, such as prophages, prophage-like elements, transposons and other integrons, and genes encoding hypothetical proteins, enzymes involved in biosynthetic pathways and TCA cycle, well characterized and putative metabolic functions, multidrug resistance efflux pumps, and fimbriae. In this study, we strived to corroborate genomic differences by demonstrating corresponding metabolic responses. Phenotype microarray results identified several substrates that *S*. Typhimurium ST221_31B was able to more effectively metablolize, as compared to *S*. Kentucky SK222_32B. For at least four of these substrates, namely D-tagatose, myo-inositol, sialic acid operon, and D-galactonate, genomic differences were also clearly seen.

Of the phenotypic differences observed, of particular interest from a virulence perspective are differences in utilizing 1, 2-propanediol, m-inositol, and N-Acetyl-D-Mannosamine substrates. *S*. Typhimurium ST221_31B was found to metabolize these substrates at a higher rate as compared to *S*. Kentucky SK222_32B. The substrate 1,2-propanediol is a major degradation product of the two commonly found sugars in plants, e.g., rhamnose and fucose. It serves as an important carbon source for organisms (i.e., *Salmonella*) that thrive under anaerobic conditions similar to that within mammalian large intestine [42–43]. The *pdu* operon codes for propanediol utilization in *Salmonella* [44]. Studies showed that the ability to degrade 1,2-propanediol has been directly linked to *Salmonella* virulence [45–47]. Although the exact mechanism relating to its virulence is unknown, deleting the *pdu* operon in some *Salmonella* strains resulted in the loss of virulence in those strains [45]. Comparative genomics indicates that the *pdu* operon is a core genome element, and phylogenetic evidence corroborates that it has evolved in a similar fashion to the whole genome of *Salmonella* serovars (“S5 Fig”). It is unclear if subtle SNP differences between serovars have resulted in loss of function or reduced function of this operon in strain SK222_32B. Another explanation could be the loss or modification of an unknown regulatory gene involved in controlling expression of this gene cluster. In contrast, comparative genomics revealed that the genome of strain SK222_32B was missing the myo-inositol utilization operon and a sialic acid utilization operon, which is presumed to account for differences in the utilization of M-inositol and N-Acetyl-D-Mannosamine, a precursor of sialic acid, respectively. We hypothesize that both phenotypic traits may be important in virulence, but mutagenesis-virulence model testing is needed to test this supposition.

The ability of a pathogen to survive phagocytosis and replicate within the phagosome is an important strategy to evade host defense mechanisms. Pathogenic *Salmonella* sp. have the ability to survive within host macrophages and this is an important aspect of their virulence [48]. In addition to macrophages, *Salmonella* Enteritidis has been reported to invade and replicate within the chicken granulosa ovarian cells [29], a model that has been used to study the egg-yolk contaminating capability. In this study, we applied both approaches and compared the invasion and intracellular survival of *S*. Typhimurium (ST221_31B) and *S*. Kentucky (SK222_32B) strains in macrophages, and in primary ovarian granulosa cells. The results of this study demonstrate that in both assays, *S*. Typhimurium ST221_31B was significantly more successful in terms of intracellular survival, with *S*. Kentucky SK222_32B more rapidly eliminated from macrophages and unable to invade and proliferate in ovarian granulosa cells. The inability to invade and replicate within the ovarian granulosa cells suggests a lack of potential for the *S*. Kentucky isolate to contaminate eggs, as compared to *S*. Typhimurium

The superoxide dismutase enzyme (SodCI), which is known to play a vital role in *Salmonella* pathogenicity by intercepting reactive oxygen species produced by the host' innate immune response [49], was only present in the genome of *S*. Typhimurium ST221_31B.

As mentioned above, one of the major steps in bacterial pathogenicity is host invasion. *S*. Typhimurium accomplishes this by sending a battery of proteins into host cell cytoplasm by way of type-III secretion systems [50–51]. One of these proteins is SopB, an inositol phosphatase, which is essential for promoting host cytoskeleton rearrangement and subsequent *Salmonella* internalization. M-inositol serves as the structural basis for many inositol phosphates including SopB [52]. Comparative genomics revealed that the gene coding for SopB protein were present in both ST221_31B and SK222_32B genomes; however, the myo-inositol catabolic pathway is not present in the genome of SK222_32B. The ability to utilize M-inositol, as well as 1,2-propanediol, by ST221_31B, as compared to SK222_32B, is one possible factor which may partly indicate that *S*. Kentucky strain SK222_32B may be unable to cause human infections. Additionally, the superoxide dismutase enzyme (SodCI), which is known to play a vital role in *Salmonella* pathogenicity by intercepting reactive oxygen species produced by the host' innate immune response [53], was only present in the genome of *S*. Typhimurium ST221_31B. More *Salmonella* strains of both Typhimurium and Kentucky serovars need to be tested for utilization of these compounds in future to confirm this trend.

In conclusion, the survival of *S*. Typhimurium ST221_31B within the macrophage cells even after 48 h and a 3-fold increase in growth in chicken ovarian granulosa cells demonstrate that *S*. Typhimurium ST221_31B has the potential to infect chickens, and subsequently can lead to human infections. We identified several differential traits between these two strains, which may account for these differences in the *in vitro* pathogenicity models used in this study. *S*. Typhimuirum ST221_31B harbored three type III secretion system effector protein-encoding genes that were missing in SK222_32B, namely, *seeI*, *sseK2*, and *sspH2*. Genome sequencing and corresponding phenotypic microarray results indicated that *S*. Typhimurium strain ST221_31B has the ability to utilize three carbon compounds, myo-inositol, sialic acid, and 1,2-propanediol, which have been linked to virulence, significantly better than *S*. Kentucky strain SK222_32B. Although, in this study, we cannot definitively point to one or several factors as direct causes for the apparent difference in virulence potential between serovars *S*. Typhimurium and *S*. Kentucky, several factors were identified as candidates for further, detailed studies, which could directly test their effect on virulence in *Salmonella enterica*. Moreover, it is important to conduct research using multiple strains of each serovar as comparison of only two MDR genomes may not necessarily be representative of the entire strain population of a certain serovar.

## Supporting information

S1 Fig The rooted phylogenetic tree based on core genome of *S*. *enterica* (A) Maximum likelihood tree, (B) Maximum parsimony tree.
(DOC)

S2 Fig Genome comparison of *Salmonella enterica* serovar Typhimurium ST221_31B with 9 other *Salmonella* genomes. (DOC)

S3 Fig Genome comparison of *Salmonella enterica* serovar Kentucky SK222_32B with 9 other *Salmonella* reference genomes. (DOC)

S4 Fig (A) Phylogenetic reconstruction of homologous Incl1 plasmid of *Salmonella enterica*, (B) BLASTN comparison of homologous *Salmonella enterica* Incl1 class plasmids.
(DOC)

S5 Fig *pdu* operon tree. (DOC)

S1 Table General feature of *S*. Typhimurium (ST221_31B) and *S*. Kentucky (SK222_32B) genomes. (DOC)

S2 Table Identification of gaps or genetic differences in *Salmonella* Typhimurium. (DOC)

S3 Table Identification of gaps or genetic differences in *Salmonella* Kentucy. (DOC)

## References

[1] Majowicz SE, Musto J, Scalla E, Angulo FJ, Kirk M, O’Brien SJ, et al The global burden of nontyphoidal *Salmonella* gastroenteritis. Clinic Infect Dis. 2010;50: 882–889. 10.1086/650733 20158401

[2] Scallan E, Hoekstra RM, Angulo FJ, Tauxe RV, Widdowson MA, Roy SL, et al Foodborne illness acquired in the United States—major pathogens. Emerg Infect Dis. 2011;17: 7–15. doi: 10.3201/eid1701.P11101 21192848 10.3201/eid1701.P11101 PMC3375761

[3] Mead PS, Slutsker L, Dietz V, McCaig LF, Bresee JS, Shapiro C, et al Food-related illness and death in the United States. Emerg Infect Dis. 1999;5: 607–625. doi: 10.3201/eid0505.990502 10511517 10.3201/eid0505.990502 PMC2627714

[4] Hoffmann S, Maculloch B, Batz M. Economic burden of major foodborne illnesses acquired in the United States. USDA Economic Research Service. 2015 Economic Information Bulletin Number 140:53.

[5] Haley BJ, Kim SW, Liljebjelke K, Guard J, Van Kessel JAS. Genome sequences of two *Salmonella enterica* serovar Kentucky isolates recovered from poultry carcasses in the United States. Genome Announc. 2016;4(6):e01289–16. doi: 10.1128/genomeA.01289-16 27856587 10.1128/genomeA.01289-16 PMC5114379

[6] Parveen S, Taabodi M, Schwarz JG, Oscar TP, Harter-Dennis J, White DG. Prevalence and antimicrobial resistance of *Salmonella* recovered from processed poultry. J Food Prot. 2007;70: 2466–2472. 18044422 10.4315/0362-028x-70.11.2466

[7] CDC. Salmonella surveillance: annual summary, 2006. U.S. Department of Health and Human Services, CDC, Atlanta, GA. 2008.

[8] USDA. Serotype profiles of Salmonella strains from meat and poultry products January 1998 through December 2011. 2013. Available: http://www.fsis.usda.gov/wps/portal/fsis/topics/data-collection-and-reports/microbiolog-y/annual-serotyping-reports. Accessed 1 December 2014.

[9] Le Hello S, Bekhit A, Granier S, Barua H, Beutlich J, Zając M, et al The global establishment of a highly-fluoroquinolone resistant *Salmonella enterica* serotype Kentucky ST198 strain. Front Microbiol 2013;4: 395 doi: 10.3389/fmicb.2013.00395 24385975 10.3389/fmicb.2013.00395 PMC3866546

[10] Le Hello S, Hendriksen RS, Doublet B, Fisher I, Nielsen EM, Whichard JM, et al International spread of an epidemic population of *Salmonella enterica* serotype Kentucky ST198 resistant to ciprofloxacin. J Infec Dis. 2011;204: 675–684. 21813512 10.1093/infdis/jir409

[11] Voetsch AC, Van Gilder TJ, Angulo FJ, Farley MM, Shallow S, Marcus R, et al Food net estimate of the burden of illness caused by nontyphoidal *Salmonella* infections in the United States. Clin Infect Dis. 2004;38: 127–134. 10.1086/381578 15095181

[12] USDA. Use of chlorine to treat poultry chiller water. Food Safety and Inspection Service, FSIS Notice 45–03. 2003.

[13] Russell SM. Controlling *Salmonella* in Poultry Production and Processing. CRC Press, Boca Raton; 2012.

[14] Sneeringer S. Restrictions on antibiotic use for production purposes in U.S. livestock industries likely to have small effects on prices and quantities. United States Department of Agriculture, Economic Research Service. 2015; Available: http://www.ers.usda.gov/amber-waves.aspx. Accessed 24 May 2016.

[15] Oscar TP, Rutto GK, Ludwig JB, Parveen S. Qualitative map of *Salmonella* contamination on young chicken carcasses. J Food Prot. 2010;73: 1596–1603. 20828464 10.4315/0362-028x-73.9.1596

[16] Mohamed T, Zhao S, White DG, Parveen S. Molecular characterization of antibiotic resistant *Salmonella* Typhimurium and *Salmonella* Kentucky isolated from pre- and post-chill whole broilers carcasses. Food Microbiol. 2014;38: 6–15. doi: 10.1016/j.fm.2013.08.002 24290620 10.1016/j.fm.2013.08.002

[17] Zhao S, McDermott PF, Friedman S, Qaiyumi S, Abbott J, Kiessling C, et al Characterization of antimicrobial-resistant *Salmonella* isolated from imported foods. J Food Prot. 2006;69: 500–507. 16541678 10.4315/0362-028x-69.3.500

[18] Aziz RK, Bartels D, Best AA, DeJongh M, Disz T, Edwards RA. The RAST Server: rapid annotations using subsystems technology. BMC Genomics. 2008;8;9: 75. 10.1186/1471-2164-9-75 PMC2265698 18261238

[19] Saitou N, Nei M. The neighbor-joining method: A new method for reconstructing phylogenetic trees. Mol Biol Evol. 1987;4: 406–425. 3447015 10.1093/oxfordjournals.molbev.a040454

[20] Tamura K, Nei M. Estimation of the number of nucleotide substitutions in the control region of mitochondrial dna in humans and chimpanzees. Mol Biol Evol. 1993;10: 512–526. 8336541 10.1093/oxfordjournals.molbev.a040023

[21] Kumar S, Stecher G, Tamura K. MEGA7: Molecular Evolutionary Genetics Analysis version 7.0 for bigger datasets. Mol Biol Evol. 2016;33:1870–1874. doi: 10.1093/molbev/msw054 27004904 10.1093/molbev/msw054 PMC8210823

[22] Treangen TJ, Ondov BD, Koren S, Phillippy AM. The Harvest suite for rapid core-genome alignment and visualization of thousands of intraspecific microbial genomes. Genome Biol. 2014;15(11): 524 doi: 10.1186/s13059-014-0524-x 25410596 10.1186/s13059-014-0524-x PMC4262987

[23] Karlin S, Altschul SF. Methods for assessing the statistical significance of molecular sequence features by using general scoring schemes. Proc Natl Acad Sci. 1990;87: 2264–2268. 2315319 10.1073/pnas.87.6.2264 PMC53667

[24] Overbeek R, Olson R, Pusch GD, Olsen GJ, Davis JJ, Disz T, et al The SEED and the Rapid Annotation of microbial genomes using Subsystems Technology (RAST). Nucleic Acids Res. 2014;42: D206–D214. doi: 10.1093/nar/gkt1226 24293654 10.1093/nar/gkt1226 PMC3965101

[25] Carattoli A, Zankari E, Garcia-Fernandez A, Voldby Larsen M, Lund O, Villa L, et al *In silico* detection and typing of plasmids using PlasmidFinder and plasmid multilocus sequence typing. Antimicrob Agents Chemother. 2014;58(7): 3895–903. doi: 10.1128/AAC.02412-14 24777092 10.1128/AAC.02412-14 PMC4068535

[26] Beug H, von Kirchbach A, Doderlain G, Conscience JF, Graf T. Chicken hematopoietic cells transformed by seven strains of defective avian leukemia viruses display three distinct phenotypes of differentiation. Cell. 1979;18: 375–390. 227607 10.1016/0092-8674(79)90057-6

[27] Bowe F, Heffron F. Isolation of *Salmonella* mutants defective for intracellular survival. Methods Enzymol. 1994;236: 509–26. 7968635 10.1016/0076-6879(94)36039-1

[28] Alam MS, Zaki MH, Yoshitake J, Akuta T, Ezaki T, Akaike T. Involvement of *Salmonella* enterica serovar Typhi RpoS in resistance to NO-mediated host defense against serovar Typhi infection. Microb Pathog. 2006;40: 116–125. doi: 10.1016/j.micpath.2005.11.007 16448800 10.1016/j.micpath.2005.11.007

[29] Thiagarajan D, Saeed M, Turek J, Asem E. *In vitro* attachment and invasion of chicken ovarian granulosa cells by *Salmonella* enteritidis phage type 8. Infect Immun. 1996;64(12): 5015–21. 8945540 10.1128/iai.64.12.5015-5021.1996 PMC174482

[30] Gilbert AB, Evans AJ, Perry MM, Davidson MH. A method for separating the granulosa cells, the basal lamina and the theca of the preovulatory ovarian follicle of the domestic fowl (Gallus domesticus). J Reprod Fertil. 1977;50: 179–181. 864645 10.1530/jrf.0.0500179

[31] Rabsch W, Andrews HL, Kingsley RA, Prager R, Tschape H, Adams LG, et al *Salmonella enterica* serotype Typhimurium and its host-adapted variants. Infect Immun. 2002;70: 2249–2255. doi: 10.1128/IAI.70.5.2249-2255.2002 11953356 10.1128/IAI.70.5.2249-2255.2002 PMC127920

[32] Fricke WF, McDermott PF, Mammel MK, Zhao S, Johnson TJ, Rasko DA, et al Antimicrobial resistance-conferring plasmids with similarity to virulence plasmids from avian pathogenic *Escherichia coli* strains in *Salmonella enterica* serovar Kentucky isolates from poultry. Appl Environ Microbiol. 2009;75: 5963–5971. doi: 10.1128/AEM.00786-09 19648374 10.1128/AEM.00786-09 PMC2747853

[33] Hoffmann M, Muruvanda T, Allard MW, Korlach J, Roberts RJ, Timme R, et al Complete genome sequence of a multidrug-resistant *Salmonella enterica* serovar Typhimurium var. 5-strain isolated from chicken breast. Genome Announc. 2013;1(6): e01068–13. doi: 10.1128/genomeA.01068-13 24356834 10.1128/genomeA.01068-13 PMC3868858

[34] Ramos-Morales F. Impact of *Salmonell*a enterica type III secretion system effectors on the eukaryotic host cell. ISRN Cell Biol. 2012;78934.

[35] Sly LM, Guiney DG, Reiner NE. *Salmonella enterica* serovar Typhimurium periplasmic superoxide dismutases SodCI and SodCII are required for protection against the phagocyte oxidative burst. Infect Immun. 2002;70: 5312–5315. doi: 10.1128/IAI.70.9.5312-5315.2002 12183590 10.1128/IAI.70.9.5312-5315.2002 PMC128279

[36] Fricke WF, Mammel MK, McDermott PF, Tartera C, White DG, LeClerc JE, et al Comparative genomics of 28 *Salmonella enterica* isolates: evidence for CRISPR-mediated adaptive sublineage evolution. J Bacteriol. 2011;193: 3556–3568. doi: 10.1128/JB.00297-11 21602358 10.1128/JB.00297-11 PMC3133335

[37] Montero I, Herrero-Fresno A, Rodicio R, Rodicio MR. Efficient mobilization of a resistance derivative of pSLT, the virulence plasmid specific of *Salmonella* enterica serovar Typhimurium, by an IncI1 plasmid. Plasmid. 2013;70: 104–109. doi: 10.1016/j.plasmid.2013.03.002 23541844 10.1016/j.plasmid.2013.03.002

[38] Sheppard AE, Stoesser N, Wilson DJ, Sebra R, Kasarskis A, Ansona LW, et al Nested Russian doll-like genetic mobility drives rapid dissemination of the carbapenem resistance gene *bla*_KPC__._ Antimicrob Agents Chemother. 2016;60: 3767*–*3778. doi: 10.1128/AAC.00464-16 27067320 10.1128/AAC.00464-16 PMC4879409

[39] Rotger R, Casadesús J. The virulence plasmids of *Salmonella*. Internatl Microbiol. 1999;2: 177–184. 10943411

[40] Chu C, Chiu C-H. Evolution of the virulence plasmids of non-typhoid *Salmonella* and its association with antimicrobial resistance. Microb Infect. 2006;8: 1931–1936. 10.1016/j.micinf.2005.12.026 16713725

[41] Rychlik I, Gregorova D, Hradecka H. Distribution and function of plasmids in *Salmonella enterica*. Vet Microbiol. 2006;112: 1–12. doi: 10.1016/j.vetmic.2005.10.030 16303262 10.1016/j.vetmic.2005.10.030

[42] Obradors N, Badia J, Baldoma L, Aguilar J. Anaerobic metabolism of the L-rhamnose fermentation product 1,2-propanediol in *Salmonella typhimurium*. J Bacteriol. 1988;170: 2159–2162. 3283105 10.1128/jb.170.5.2159-2162.1988 PMC211101

[43] Cheng S, Sinha S, Fan C, Liu Y, Bobik TA. Genetic analysis of thhe protein shell of the microcompartments involved in coenzyme B12-dependent 1,2-propanediol degradation by *Salmonella*. J Bacteriol. 2011;193(6): 1385–1392. doi: 10.1128/JB.01473-10 21239588 10.1128/JB.01473-10 PMC3067621

[44] Bobik TA, Havemann GD, Busch RJ, Williams DS, Aldrich HC. The propanediol utilization (*pdu*) operon of *Salmonella enterica* serovar Typhimurium LT2 includes genes necessary for formation of polyhedral organelles involved in coenzyme B12-dependent 1,2-propanediol degradation. J Bacteriol. 1999;181(19): 5967–5975. 10498708 10.1128/jb.181.19.5967-5975.1999 PMC103623

[45] Conner CP, Heithoff DM, Julio SM, Sinsheimer RL, Mahan MJ. Differential patterns of acquired virulence genes distinguish *Salmonella* strains. Proc Natl Acad Sci USA. 1998;95: 4641–4645. 9539791 10.1073/pnas.95.8.4641 PMC22543

[46] Thiennimitr P, Winter SE, Winter MG, Xavier MN, Tolstikov V, Huseby DL, et al Intestinal inflammation allows *Salmonella* to use ethanolamine to compete with the microbiota. Proc Natl Acad Sci USA. 2011;108(42): 17480–17485. doi: 10.1073/pnas.1107857108 21969563 10.1073/pnas.1107857108 PMC3198331

[47] Winter SE, Baumler AJ. A breathtaking feat: to compete with the gut microbiota, *Salmonella* drives its host to provide a respiratory electron acceptor. Gut Microbes. 2011;2: 58–60. doi: 10.4161/gmic.2.1.14911 21637020 10.4161/gmic.2.1.14911 PMC3225798

[48] Lindgren SW, Stojiljkovic I, Heffron F. Macrophage killing is an essential virulence mechanism of *Salmonella* typhimurium. Proc Natl Acad Sci USA. 1996;93: 4197–4201. 8633040 10.1073/pnas.93.9.4197 PMC39511

[49] Uzzau S, Figueroa-Bossi N, Rubino S, Bossi L. Epitope tagging of chromosomal genes in *Salmonella*. Proc Natl Acad Sci USA. 2001;98: 15264–15269. doi: 10.1073/pnas.261348198 11742086 10.1073/pnas.261348198 PMC65018

[50] Patel JC, Galan JE. Manipulation of the host actin cytoskeleton by *Salmonella*- all in the name of entry. Curr Opin Microbiol. 2005;8: 10–15. doi: 10.1016/j.mib.2004.09.001 15694851 10.1016/j.mib.2004.09.001

[51] McGhie EJ, Brawn LC, Hume PJ, Humphreys D, Koronakis V. *Salmonella* takes control: effector-driven manipulation of the host. Curr Opin Microbiol. 2009;12: 117–124. doi: 10.1016/j.mib.2008.12.001 19157959 10.1016/j.mib.2008.12.001 PMC2647982

[52] Zhou D, Chen L, Hernandez L, Shears SB, Galan JE. A *Salmonella* inositol polyphosphatase acts in conjunction with other bacterial effectors to promote host cell actin cytoskeleton rearrangements and bacterial internalization. Mol Microbiol. 2001;39(2): 248–259. 11136447 10.1046/j.1365-2958.2001.02230.x

[53] Uzzau S, Figueroa-Bossi N, Rubino S, Bossi L. Epitope tagging of chromosomal genes in *Salmonella*. Proc Natl Acad Sci USA. 2001;98: 15264–15269. doi: 10.1073/pnas.261348198 11742086 10.1073/pnas.261348198 PMC65018

