# Supplementary material for: Genotypic and phenotypic characterization of multidrug resistant Salmonella Typhimurium and Salmonella Kentucky strains recovered from chicken carcasses
Source: PLoS One. 2017 May 8;12(5):e0176938. doi: 10.1371/journal.pone.0176938 (PMC5421757; doi:10.1371/journal.pone.0176938)
Supplement: S1 Fig — The rooted phylogenetic tree based on core genome of S. enterica (A) Maximum likelihood tree, (B) Maximum parsimony tree. (DOC) [file pone.0176938.s001.doc]

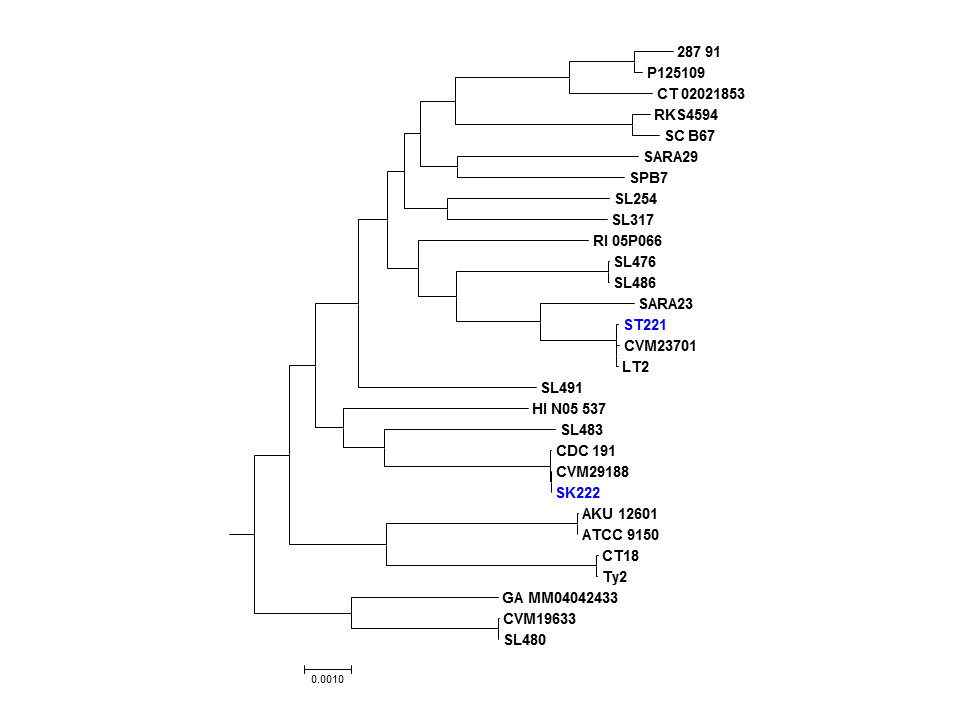


**S1Fig. The rooted phylogenetic tree based on core genome of *S. enterica* (A)** Maximum likelihood tree was constructed based on homologous alignment of 1,185 conserved ORFs (1,113,790 bp). The tree was rooted with *Salmonella enterica* subsp. arizonae serovar 62:z4,z23:- str. RSK2980. Bar represents 0.001 substitutions per site.


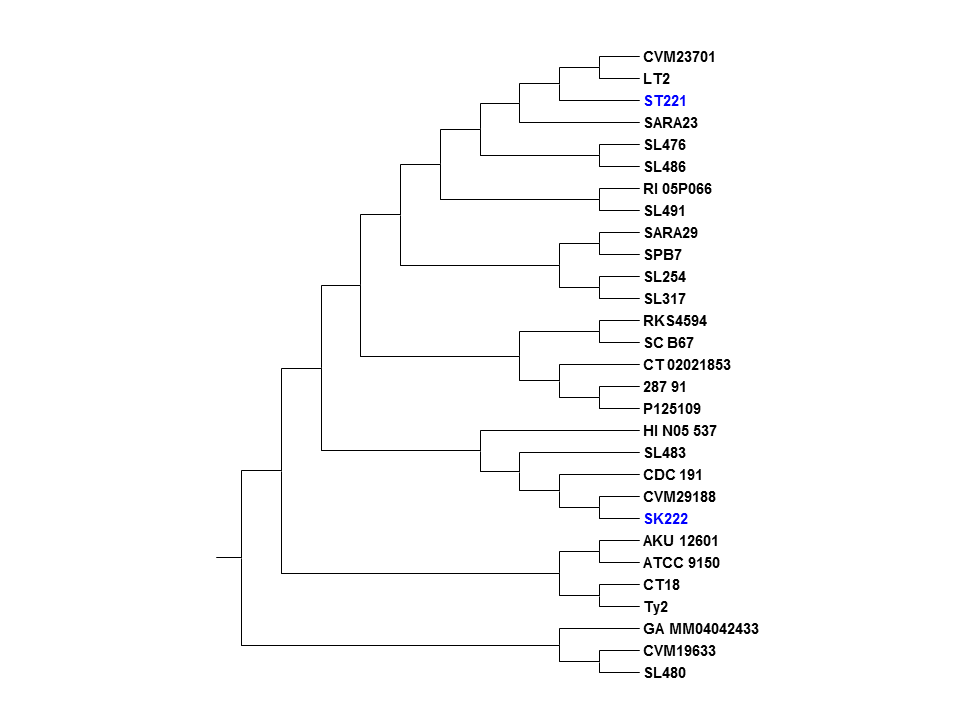


**S1Fig. The rooted phylogenetic tree based on core genome of *S. enterica* (B)** Maximum parsimony tree was constructed based on homologous alignment of 1,185 conserved ORFs (1,113,790 bp). The tree was rooted with *Salmonella enterica* subsp. arizonae serovar 62:z4,z23:- str. RSK2980. Bar represents 0.001 substitutions per site.
