# Supplementary material for: Genotypic and phenotypic characterization of multidrug resistant Salmonella Typhimurium and Salmonella Kentucky strains recovered from chicken carcasses
Source: PLoS One. 2017 May 8;12(5):e0176938. doi: 10.1371/journal.pone.0176938 (PMC5421757; doi:10.1371/journal.pone.0176938)
Supplement: S2 Fig — (DOC) [file pone.0176938.s002.doc]

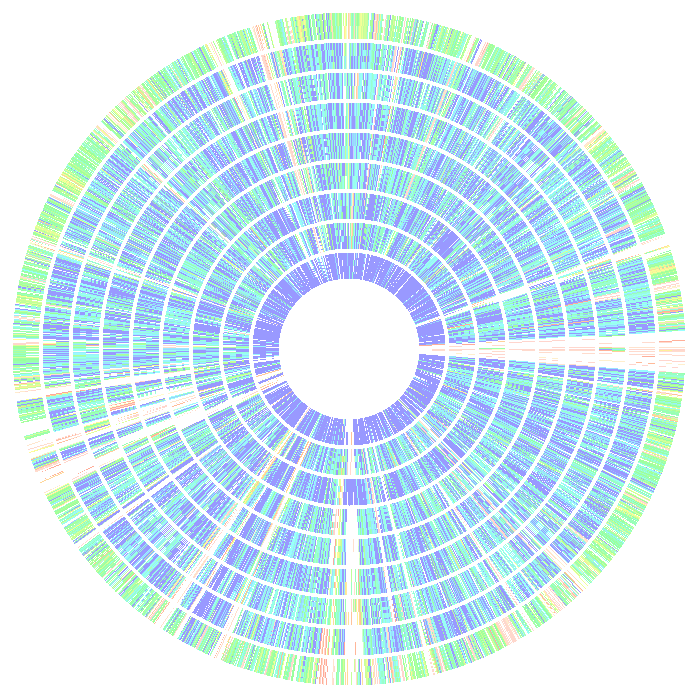


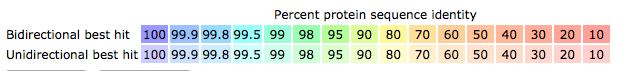


**S2 Fig. Genome comparison of *Salmonella enterica* serovar Typhimurium ST221_31B with 9 other *Salmonella* genomes.** From outer to innermost rings represent genomes of *Salmonella* reference strains - RKS2980, SL483, SCB67, CT_02021853, P125109, 287/91, SL476, CVM29188, LT2, respectively. Arrow indicates areas of genomic differences of mobile elements.
