# Supplementary material for: Genotypic and phenotypic characterization of multidrug resistant Salmonella Typhimurium and Salmonella Kentucky strains recovered from chicken carcasses
Source: PLoS One. 2017 May 8;12(5):e0176938. doi: 10.1371/journal.pone.0176938 (PMC5421757; doi:10.1371/journal.pone.0176938)
Supplement: S4 Fig — (A) Phylogenetic reconstruction of homologous Incl1 plasmid of Salmonella enterica, (B) BLASTN comparison of homologous Salmonella enterica Incl1 class plasmids. (DOC) [file pone.0176938.s004.doc]

**A**


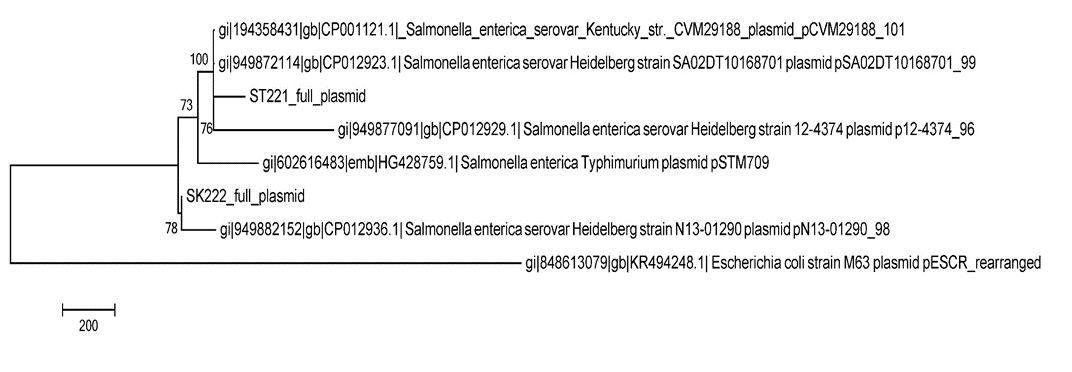


**B**
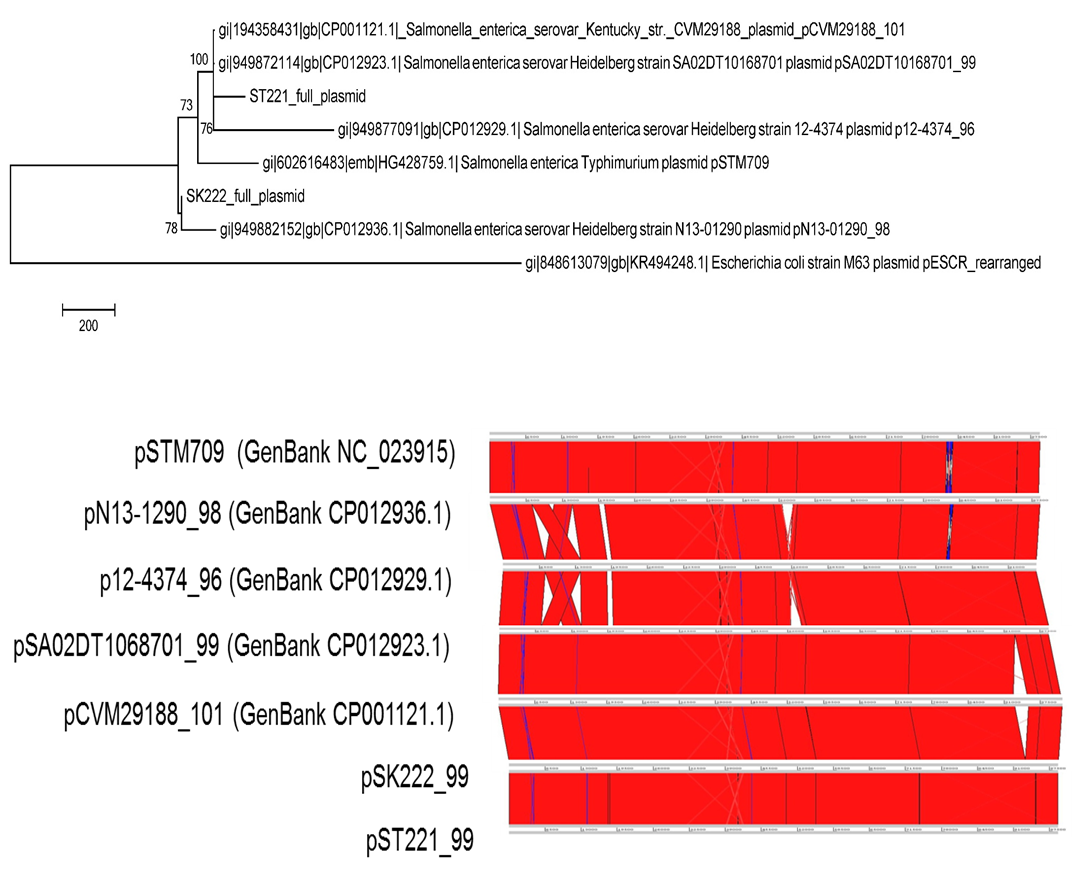


**S4 Fig. (A) Phylogenetic reconstruction of homologous Incl1 plasmid of *Salmonella enterica*, (B) BLASTN comparison of homologous *Salmonella enterica* Incl1 class plasmids.**
