# Supplementary figures and images for: Genotypic and phenotypic characterization of multidrug resistant Salmonella Typhimurium and Salmonella Kentucky strains recovered from chicken carcasses
Source: PLoS One. 2017 May 8;12(5):e0176938. doi: 10.1371/journal.pone.0176938 (PMC5421757; doi:10.1371/journal.pone.0176938)

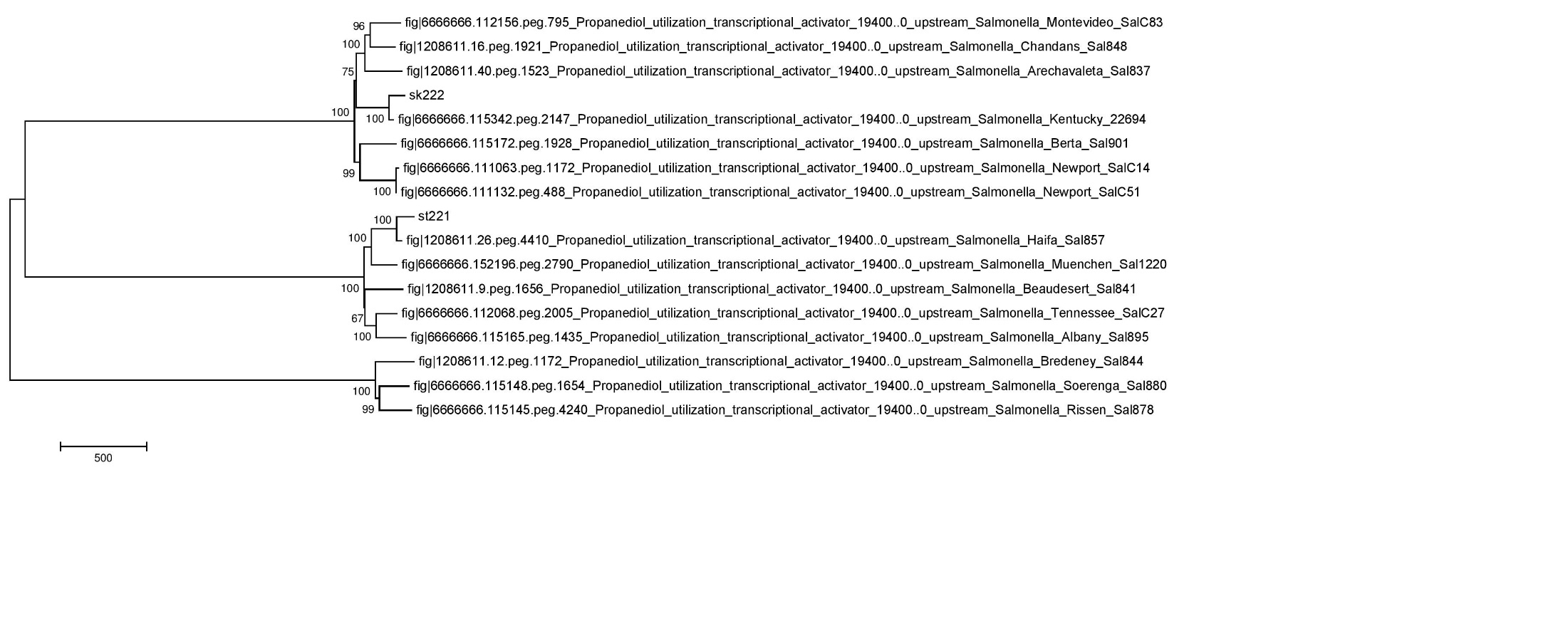


**S5 Fig. *pdu* operon tree.**

Supplement: S5 Fig — (DOC) [file pone.0176938.s005.doc]
