# Supplementary material for: Genotypic and phenotypic characterization of multidrug resistant Salmonella Typhimurium and Salmonella Kentucky strains recovered from chicken carcasses
Source: PLoS One. 2017 May 8;12(5):e0176938. doi: 10.1371/journal.pone.0176938 (PMC5421757; doi:10.1371/journal.pone.0176938)
Supplement: S1 Table — (DOC) [file pone.0176938.s006.doc]

**S1 Table.** **General feature of *S.* Typhimurium (ST221_31B) and *S.* Kentucky (SK222_32B) genomes.**

| Strains | Genome assembly | Genome Size (bp) | No. of contigs | GC Content (%) | No. of tRNAs | Accession |
| --- | --- | --- | --- | --- | --- | --- |
| ST221_31B | CLC Genomics | 4,989,491 | 72 | 52.1 | 123 | JUIT00000000 |
| SK222_32B | CLC Genomics | 5,005,607 | 55 | 51.9 | 154 | JUIU00000000 |
