# Supplementary material for: Genotypic and phenotypic characterization of multidrug resistant Salmonella Typhimurium and Salmonella Kentucky strains recovered from chicken carcasses
Source: PLoS One. 2017 May 8;12(5):e0176938. doi: 10.1371/journal.pone.0176938 (PMC5421757; doi:10.1371/journal.pone.0176938)
Supplement: S2 Table — (DOC) [file pone.0176938.s007.doc]

**S2 Table.** **Identification of gaps or genetic differences in *Salmonella* Typhimurium.**

| **Contig** | **Start (bp)** | **End (bp)** | **Best Matches** | **% G+C** | **Genes/Function** |
| --- | --- | --- | --- | --- | --- |
| 3 | 59264 | 66076 | *S*. Typhimurium, Paratyphi C, Choleraesuis, Cubana | 49 | LysR-family transcriptional regulator SinR, hypothetical protein, Polysaccharide deacetylase, Putative fimbrial structural subunit, FIG034929: Fimbriae usher protein SafC, Periplasmic fimbrial chaperone protein (*safABCD*) |
| 3, phage, inserted between tRNA-thr-CGT and tRNA-Leu-TAA | 1106 | 39935 | Bacteriophage P22 | 47.4 | Polymyxin resistance protein ArnC, glycosyl transferase, O-antigen conversion: translocase, Phage tail fibers, Phage DNA transfer protein, Phage capsid and scaffold, DNA stabilization, phage-associated, Phage portal protein, Phage terminase, large subunit and small subunit, Orf80, Phage endopeptidase, Probable lysozyme from lambdoid prophage DLP12, Phage holin, class II, Phage antitermination protein Q,  Phage Nin protein, Phage NinY, Phage NinG rap recombination,  Phage NinF, NinX, Phage NinB, Phage replicative DNA helicase,  Origin specific replication initiation factor, cII, Phage repressor protein C2, superinfection exclusion, antirestriction protein, DNA recombinase, phage-associated, essential recombination function protein Erf, Phage eae protein, unknown protein encoded within prophage CP-933RPhage EaA, EaG, EaC proteins |
| 26 | 1 | 7805 | *S*. Typhimurium | 48.9 | glycerol dehyrdratase activator, Glycerol dehydratase reactivation factor large subunit, FIG074102: hypothetical protein, putative permease, 3-isopropylmalate dehydratase large subunit (EC 4.2.1.33), 3-isopropylmalate dehydratase small subunit (EC 4.2.1.33), Fumarylacetoacetate hydrolase family protein, Putative hydrolase or acyltransferase, Putative LysR family transcriptional regulator |
| 10 | 129432 | 136843 | *S*. Typhimurium, *S*. Heidelberg, *S*. Anatum, *S*. Tennessee, *S*. Newport, *S*. Weltevreden | 54.2 | PTS system, mannose-specific IIA component (EC 2.7.1.69), PTS system, mannose-specific IIB component (EC 2.7.1.69), PTS system, mannose-specific IIC component (EC 2.7.1.69), PTS system, mannose-specific IID component (EC 2.7.1.69), Glucosamine--fructose-6-phosphate aminotransferase [isomerizing] (EC 2.6.1.16), Putative phosphosugar isomerase, NtrC family Transcriptional regulator, ATPase domain |
| 10 | 40733 | 49181 | *S*. Typhimurium, *S*. Enteritidis | 52.3 | putative cytoplasmic protein, Chaperone protein DnaK, FIG00638911: hypothetical protein, FIG00637867: hypothetical protein, Putative molecular chaperone, DnaJ family, FIG00637867: hypothetical protein, TETRATRICOPEPTIDE REPEAT FAMILY PROTEIN |
| 25 | 59075 | 63710 | *S*. Typhimurium, *S*. Heidelberg, *S*. Enteritidis | 38.6 | L(+)-tartrate dehydratase alpha subunit (EC 4.2.1.32), Transcriptional regulator, lysR family, LysR-family transcriptional regulator STM0764, Putative membrane protein, Oxaloacetate decarboxylase gamma chain (EC 4.1.1.3) |
| 25 | 15188 | 27628 | *S*. Typhimurium, *S*. Heidelberg, *S*. Anatum, *S*. Paratyphi B, *S*. Newport, *S*. Weltevreden | 38.1 | hypothetical protein, Probable DNA recombinase, putative inner membrane protein, FIG074102: hypothetical protein, UDP-galactopyranose mutase (EC 5.4.99.9), Putative glycosyl transferase  Putative glycosyl transferase, O-antigen export system permease protein RfbD, O-antigen/lipopolysaccharide transport ATP-binding protein ABC transporter RfbE, putative glycosyltransferase, FIG01045205: hypothetical protein, Dolichol-phosphate mannosyltransferase (EC 2.4.1.83) in lipid-linked oligosaccharide synthesis cluster, hypothetical protein |
| 17 | 106166 | 108192 (end) | *S*. Typhimurium, *S*. Heidelberg, *S*. Enteritidis | 43.1 | Putative inner membrane protein, Putative inner membrane protein, Putative inner membrane protein, Putative membrane protein |
| 17 | 48710 | 56132 | *S*. Typhimurium, *S*. Heidelberg, *S*. Anatum, *S*. Tennessee, *S*. Newport, *S*. Weltevreden | 41.6 | D-beta-hydroxybutyrate permease, Putative LysR family transcriptional regulator, Electron transfer flavoprotein-ubiquinone oxidoreductase (EC 1.5.5.1), Probable acyl-CoA dehydrogenase (EC 1.3.99.3), FIG01047477: hypothetical protein, Electron transfer flavoprotein, alpha subunit, Electron transfer flavoprotein, beta subunit, putative cytoplasmic protein |
| 31, phage | 58458 | 76741 | *S*. Typhimurium | 48.9 | Integrase, Phage excisionase, FIG00643583: hypothetical protein, Gifsy-2 prophage RecT, Exodeoxyribonuclease VIII (EC 3.1.11.-), FIG01045596: hypothetical protein, Phage protein, Phage CI-like repressor, putative DNA-binding protein, hypothetical protein, Origin specific replication initiation factor #replication protein O, Bacteriophage-encoded homolog of DNA replication protein DnaC, putative DNA-binding protein, Phage protein, FIG01046901: hypothetical protein, Phage EaA protein, hypothetical protein, hypothetical protein, DNA-damage-inducible protein I, hypothetical protein, Phage protein, FIG01045325: hypothetical protein, Protein NinG, antiterminator-like protein, FIG00545237: hypothetical protein |
| 22 | 181807 | 201050 | *S*. Typhimurium, *S*. Newport | 50.6 | Virulence protein msgA, FIG01046696: hypothetical protein, hypothetical protein, Gifsy-2 prophage protein, Transposase, Secreted effector protein, Phage tail fibers, Phage tail fiber protein, FIG01046172: hypothetical protein, Phage tail fiber protein, Phage tail assembly protein I, Phage tail assembly protein, Phage minor tail protein, Superoxide dismutase [Cu-Zn] precursor (EC 1.15.1.1), Attachment invasion locus protein precursor, Phage minor tail protein, Phage tail length tape-measure protein 1, Phage terminase, large subunit, Terminase small subunit, hypothetical protein, Phage outer membrane lytic protein Rz; Endopeptidase (EC 3.4.-.-), Phage lysin (EC 3.2.1.17) # Phage lysozyme or muramidase (EC 3.2.1.17), Phage holing, GtgA |
| 22 (inserted at tRNA-Ser-GGA) | 101482 | 110245 | *S*. Typhimurium, *S*. Thompson, *S*. Anatum, *S*. Enteritidis, *S*. Newport | 44 | FIG00553873: hypothetical protein, Sialic acid transporter (permease) NanT, N-acetylneuraminic acid outer membrane channel protein NanC, Sialic acid-induced transmembrane protein YjhT(NanM), possible mutarotase, N-acetylmannosamine-6-phosphate 2-epimerase (EC 5.1.3.9), Predicted sialic acid transporter, Sialic acid utilization regulator, RpiR family |
| 7 | 277352 | 279339 | *S*. Typhimurium, *S*. Newport,  *S*. Weltevreden, *S*. Thompson, *S*. Agona, *S*. Heidelberg, *S*. Anatum | 47.6 | Choloylglycine hydrolase (EC 3.5.1.24), Hexuronate utilization operon transcriptional repressor ExuR, L-galactonate dehydrogenase (EC 1.1.1.-) |
| 7 | 283428 | 288413 | *S*. Typhimurium, *S*. Thompson,  *S*. Weltevreden, *S*. Anatum, *S*. Heidelberg, *S*. Enteritidis | 47.4 | 12-TMS multidrug efflux protein homolog, Oxygenase-like protein, Transcriptional regulator, MarR family, S-adenosylmethionine:tRNA ribosyltransferase-isomerase (EC 5.-.-.-), RidA/YER057c/UK114 superfamily, group 7, YjgH-like protein |
| 7 | 288584 | 292154 | *S*. Typhimurium, *S*. Heidelberg, *S*. Anatum | 45.8 | RelE/StbE replicon stabilization toxin, RelB/StbD replicon stabilization protein, Putative phage-related secreted protein, Hypothetical protein, Putative cytoplasmic protein, Putative transposase, Putative coiled-coil protein |
| 7 | 310914 | 318804 | *S*. Typhimurium, *S*. Thompson,  *S*. Enteritidis | 51.0 | Formate dehydrogenase N gamma subunit (EC 1.2.1.2), Formate dehydrogenase N beta subunit (EC 1.2.1.2), Formate dehydrogenase N alpha subunit (EC 1.2.1.2) @ selenocysteine-containing, Permease of the drug/metabolite transporter (DMT) superfamily, Outer membrane porin protein NmpC precursor, Gfa-like protein |
| 7 | 375279 | 382887 | *S*. Typhimurium, *S*. Thompson, *S*. Anatum, *S*. Bareilly, *S*. Enteritidis, *S*. Newport | 49.9 | FIG074102: hypothetical protein, LysR family transcriptional regulator YdcI, Methyl-accepting chemotaxis protein III (ribose and galactose chemoreceptor protein), S-(hydroxymethyl)glutathione dehydrogenase (EC 1.1.1.284), FrmR: Negative transcriptional regulator of formaldehyde detoxification operon, hypothetical protein, FIG01045674: hypothetical protein, FIG01045328: hypothetical protein |
| 7 | 384660 | 390446 | *S*. Typhimurium, *S*. Heidelberg, *S*. Anatum, *S*. Newport, *S*. Thompson, *S*. Enteritidis | 41.5 | Putative inner membrane protein, Putative periplasmic binding protein, Putative ABC amino acid transporter permease, ABC transporter ATP-binding subunit, putative amino acid ABC transporter, putative membrane protein |
| 7 | 617575 | 629942 | *S*. Typhimurium, *S*. Heidelberg,  *S*. Infantis, *S*. Dublin, *S*. Newport, *S*. Enteritidis | 45.6 | FIG01046381: hypothetical protein, FIG074102: hypothetical protein, Mobile element protein, Mobile element protein, Phage integrase  hypothetical protein, hypothetical protein, Permease of the drug/metabolite transporter (DMT) superfamily, FIG01045658: hypothetical protein, hypothetical protein, hypothetical protein, hypothetical protein, Phage tail fiber protein, Phage protein, FIG01046582: hypothetical protein, lytic enzyme, hypothetical protein, phage-tail assembly-like protein, FIG01047716: hypothetical protein, Mobile element protein |
| 19, phage, adjacent to tRNA-Ser-CGA | 1 | 12702 | *S*. Typhimurium, *S*. Enteritidis, *Escherichia coli* | 49.3 | DNA methyl transferase phage-associated, Primosomal protein I, Putative antirepressor, Phage protein, Phage DNA-binding protein, Regulatory protein, Translation elongation factors (GTPases), Hypothetical protein yfdR, Phage EaA protein, Phage integrase, Integrase, Transposase, Putative periplasmic protein, AMP nucleosidae, Putative acyl carrier protein |
| 46 | 34365 | 59771 (end) |  | 51.8 | hypothetical protein, FIG00640097: hypothetical protein, Phage tail fibers, Phage tail fibers, FIG121501: Prophage tail protein, Phage FluMu protein gp47, Bacteriophage protein GP46, Prophage baseplate assembly protein V, FIG003269: Prophage tail protein, Phage tail/DNA circulation protein, Phage tail length tape-measure protein, hypothetical protein, Phage tail tube protein, Bacteriophage tail sheath protein, Mu-like prophage FluMu protein GP38, FIG111678: IS, phage, Tn; Transposon-related functions, Phage protein, Sb9, FIG01046441: hypothetical protein, FIG069533: Putative bacteriophage protein, Phage major capsid protein, Gene Transfer Agent prohead protease ## ORFG04, Phage portal protein, FIG00640168: hypothetical protein, Phage terminase, large subunit, Phage terminase, small subunit, Phage holing, probable bacteriophage protein STY2043, FIG101079: Lytic enzyme, Putative prophage membrane protein, FIG01047076: hypothetical protein, Phage antitermination protein Q, Phage antitermination protein Q |
| 34, PLE inserted next to tRNA-Pro-GGG | 1 | 9464 | *S*. Typhimurium, *S*. Heidelberg,  *S*. Paratyphi B | 44.7 | Phage antitermination protein Q, Antitermination protein Q, FIG01045701: hypothetical protein, Phage portal protein, Membrane proteins related to metalloendopeptidases, FIG01047264: hypothetical protein, FIG01047148: hypothetical protein, FIG01046717: hypothetical protein, O-antigen acetylase, hypothetical protein, virulence protein, Phage tail fiber assembly protein |
| 15 | 26740 | 29917 | *S*. Typhimurium, *S*. Typhi, *S*. Anatum, *S*. Newport, *S*. Weltevreden, *S*. Thompson | 45.2 | Transcriptional regulator, LysR family, Putative transmembrane transport protein, 2-dehydropantoate 2-reductase (EC 1.1.1.169) |
| 35 (adjacent to T1SS) | 1 | 21143 | *S.* Typhimurium strain CFSAN001921 | 50 | YeeU protein (antitoxin to YeeV), YeeV toxin protein, YfjZ protein (antitoxin to YpjF), Uncharacterized protein YkfH, DNA repair protein RadC, Antirestriction protein klcA, Puative intergenic transcriptional regulator, NgrB, Type III restriction-modification system methylation subunit, DNA-binding protein, Prophage CP4-57 regulatory protein alpA, Mobile element protein, Type I secretion system, membrane fusion protein LapC, Type I secretion system ATPase, LssB family LapB,  Type I secretion system, outer membrane component LapE, Prophage CP4-57 regulatory protein alpA, Mobile element protein, hypothetical protein, Mobile element protein |
| 54 | 1190 | 8658 (end) | *S*. Typhimurium, *S*. Heidelberg | 50.6 | Phage integrase, Phage P4-associated, integrase-like protein, putative periplasmic protein, putative cytoplasmic protein, putative cytoplasmic protein, putative cytoplasmic protein, putative inner membrane protein, hypothetical protein |
| 8 | 1 | 25275 (end) | *S*. Typhimurium, *S*. Heidelberg | 45.3 | putative ATPase  putative cytoplasmic protein  FIG01047293: hypothetical protein, FIG01047227: hypothetical protein, putative cytoplasmic protein, PTS system, glucitol/sorbitol-specific IIC component (EC 2.7.1.69), PTS system, glucitol/sorbitol-specific IIA component (EC 2.7.1.69), PTS system, glucitol/sorbitol-specific IIB component and second of two IIC components (EC 2.7.1.69), Oxidoreductase, Gfo/Idh/MocA family, FIG01047465: hypothetical protein, D-arabino-3-hexulose 6-phosphate formaldehyde lyase, 6-phospho-3-hexuloisomerase, Glucose-6-phosphate isomerase (EC 5.3.1.9), PTS system, glucose-specific IIC component (EC 2.7.1.69) / PTS system, glucose-specific IIB component (EC 2.7.1.69) / PTS system, glucose-specific IIA component (EC 2.7.1.69), putative dipeptide/oligopeptide/nickel ABC-type transport system periplasmic component, hypothetical protein, hypothetical protein, hypothetical protein, FIG01046935: hypothetical protein, hypothetical protein, putative inner membrane protein, FIG01047407: hypothetical protein, Mobile element protein, Mobile element protein, Mobile element protein, putative cytoplasmic protein, FIG01046577: hypothetical protein, Mobile element protein |
| 6 | 138308 | 146438 | *S*. Typhimurium, *S*. Heidelberg, *S*. Anatum, *S*. Bareilly, *S*. Enteritidis, *S*. Newport, *S*. Tennessee | 47.9 | FIG01048344: hypothetical protein, Putative mannitol dehydrogenase, (R)-2-hydroxyacid dehydrogenase, similar to L-sulfolactate dehydrogenase (EC 1.1.1.272), Sorbitol dehydrogenase (EC 1.1.1.14), D-mannonate oxidoreductase (EC 1.1.1.57), Muconolactone isomerase (EC 5.3.3.4),putative, hypothetical protein, Hexuronate utilization operon transcriptional repressor ExuR, Putative outer membrane lipoprotein |
| 6 | 174481 | 181234 | *S*. Typhimurium, *S*. Heidelberg, *S*. Paratyphi B, *S*. Infantis, *S*. Enteritidis, *S*. Newport | 48.7 | 4-hydroxybutyrate:acetyl-CoA CoA transferase (EC 2.3.1.-), Acyl dehydratase, Hydroxymethylglutaryl-CoA lyase (EC 4.1.3.4), LysR family transcriptional regulator STM3121, Arylsulfatase (EC 3.1.6.1), Putative arylsulfatase regulator |
| 36 | 5879 | 12030 | *S*. Typhimurium, *S*. Thompson, *S*. Paratyphi A, *S*. Typhi | 47.9 | Fructokinase (EC 2.7.1.4), Transcriptional repressor of aga operon, Tagatose 1,6-bisphosphate aldolase (EC 4.1.2.40), Tagatose-6-phosphate kinase (EC 2.7.1.144) / 1-phosphofructokinase (EC 2.7.1.56), PTS system, galactose-inducible IIB component (EC 2.7.1.69) / PTS system, galactose-inducible IIC component (EC 2.7.1.69), PTS system, tagatose-specific IIA-TPr component (EC 2.7.1.69) |
| 36 | 103381 | 104637 (end) | *S*. Typhimurium, *S*. Enteritidis | 57.2 | Membrane protein associated with oxaloacetate decarboxylase, Oxaloacetate decarboxylase beta chain (EC 4.1.1.3) |
| 32 | 40492 | 461181 (end) | *S*. Typhimurium, *S*. Heidelberg, *S*. Anatum, *S*. Thompson, *S*. Enteritidis | 52.4 | Oxaloacetate decarboxylase gamma chain (EC 4.1.1.3), L(+)-tartrate dehydratase beta subunit (EC 4.2.1.32), L(+)-tartrate dehydratase alpha subunit (EC 4.2.1.32), Putative membrane protein, Transcriptional regulator, GntR family, Transcriptional regulator, GntR family |
| 30 | 43949 | 50707 | *S*. Typhimurium, *S*. Heidelberg, *S*. Enteritidis | 56.7 | YafQ toxin protein, DNA-damage-inducible protein J, RNA 3'-terminal phosphate cyclase (EC 6.5.1.4), RNA-2',3'-PO4:RNA-5'-OH ligase, Putative ribonucleoprotein related-protein, Putative ribonucleoprotein related-protein, hypothetical protein, Transcriptional regulatory protein RtcR |
| 30 | 54542 | 62991 | *S*. Typhimurium, *S*. Heidelberg, , *S*. Thompson, *S*. Enteritidis | 51.3 | Putative inner membrane protein, Phosphate ABC transporter, periplasmic phosphate-binding protein PstS (TC 3.A.1.7.1), Glycerol dehydrogenase (EC 1.1.1.6), Nitrate/nitrite transporter, Dihydroxy-acid dehydratase (EC 4.2.1.9), 4-hydroxy-tetrahydrodipicolinate synthase (EC 4.3.3.7), Transcriptional regulator, ArsR family |
| 30 | 79873 | 84673 | *S*. Typhimurium, *S*. Heidelberg, , *S*. Anatum, *S*. Enteritidis | 47.0 | Putative acetyltransferase, hypothetical protein, Putative ribokinase, FIG074102: hypothetical protein, Putative inner membrane protein, Puative phophotriesterase |
| 30 | 116090 | 121019 | *S*. Typhimurium, *S*. Thompson, *S*. Weltevreden, *S*. Paratyphi A, *S*. Newport, *S*. Agona | 58.1 | FIG01045128: hypothetical protein, ABC-type multidrug transport system, permease component, Putative membrane protein |
| 30 (adjacent to tRNA-Pro-CGG) | 180844 | 184508 | *S*. Typhimurium, *S*. Heidelberg, , *S*. Newport, *S*. Weltevreden, *S*. Enteritidis | 56.6 | Xanthine permease, Putative PQQ enzyme repeat, hypothetical protein, Putative lacI-family transcriptional regulator |
| 30 (*lpfABCDE*) | 186744 | 192398 (end) | *S*. Typhimurium, *S*. Heidelberg, *S*. Newport, *S*. Thompson, *S*. Enteritidis | 50.1 | putative fimbrial protein precursor, Putative fimbrial protein, type 1 fimbriae anchoring protein FimD, Chaperone protein lpfB precursor, Long polar fimbria protein A precursor |
| 24 | 59286 | 63561 | *S*. Typhimurium, *S*. Heidelberg, *S*. Enteritidis | 46.6 | hypothetical protein, FIG01046176: hypothetical protein, ATP binding protein, FIG01046146: hypothetical protein, Putative DNA-binding protein in cluster with Type I restriction-modification system, FIG01046502: hypothetical protein |
| 24 | 27159 | 44527 | *S*. Typhimurium, *S*. Heidelberg, *S*. Anatum, *S*. Thompson, *S*. Enteritidis | 51.4 | 4-hydroxy-2-oxoglutarate aldolase (EC 4.1.3.16), L-seryl-tRNA(Sec) selenium transferase-related protein, PTS system, mannose-specific IID component (EC 2.7.1.69), PTS system, mannose-specific IIC component, PTS system, gluconate-specific IIB component (EC 2.7.1.69), PTS system, mannose-specific IIA component, Transcriptional regulatory protein zraR, hypothetical protein, Putative secreted protein, hypothetical protein, Beta-glucosidase (EC 3.2.1.21), Putative transport protein, FIG074102: hypothetical protein, Putative DNA-binding protein in cluster with Type I restriction-modification system, Phosphotransferase system HPr enzyme STM3779, Fructose-bisphosphate aldolase (EC 4.1.2.13), Putative carbohydrate kinase in cluster with fructose-bisphosphate aldolase, Putative carbohydrate PTS system, IIC component (EC 2.7.1.69), Putative carbohydrate PTS system, IIB component (EC 2.7.1.69), Putative carbohydrate PTS system, IIA component (EC 2.7.1.69), Putative transcriptional regulator of unknown carbohydrate utilization cluster, GntR family |
| 27 | 10643 | 15375 | *S*. Typhimurium, *S*. Heidelberg, *S*. Newport, *S*. Thompson, *S*. Anatum, *S*. Enteritidis | 55.3 | D-galactonate transporter, Galactonate dehydratase (EC 4.2.1.6), 2-dehydro-3-deoxyphosphogalactonate aldolase (EC 4.1.2.21), 2-dehydro-3-deoxygalactonokinase (EC 2.7.1.58), D-Galactonate repressor DgoR |
| 45 | 17856 | 22547 | *S*. Typhimurium, *S*. Agona, *S*. Enteritidis | 48.4 | Putative hydrolase, FIG01046022: hypothetical protein, Hypothetical radical SAM family enzyme in interesting gene cluster, FIG01124638: hypothetical protein, FIG00638667: hypothetical protein |
| 29 | 18692 | 26511 | *S*. Typhimurium, *S*. Thompson, *S*. Newport, *S*. Enteritidis | 55.8 | Phosphoenolpyruvate-protein phosphotransferase of PTS system (EC 2.7.3.9), PTS system, fructose-specific IIC component, PTS system fructose-like IIB component 1 precursor (EC 2.7.1.69), Pyruvate formate-lyase (EC 2.3.1.54), Pyruvate formate-lyase activating enzyme (EC 1.97.1.4), PTS system fructose-like IIB component 2 precursor (EC 2.7.1.69) |
| 50 | 12273 | 33895 | *S*. Typhimurium, *S*. Heidelberg, *S*. Anatum, *S*. Agona, *S*. Newport, *S*. Enteritidis | 50.8 | Sodium-dependent transporter, FIG01046261: hypothetical protein, putative cytoplasmic protein, putative inner membrane protein, hypothetical protein, putative cytoplasmic protein, putative cytoplasmic protein, putative phage tail fiber protein H, Putative phage tail protein, Phage baseplate, putative bacteriophage baseplate protein, hypothetical protein, putative inner membrane protein, Polymyxin resistance protein ArnC, glycosyl transferase (EC 2.4.-.-), putative phage glucose translocase, hypothetical protein, Putative phage baseplate component, Gene D protein, Putative inner membrane protein, FIG00639790: hypothetical protein, Phage tail length tape-measure protein, hypothetical protein, FIG00639134: hypothetical protein, Putative phage tail core protein, Phage tail sheath monomer, FIG074102: hypothetical protein, Phage protein, Putative inner membrane protein, Phage lysin, Putative inner membrane protein, putative cytoplasmic protein |
| 23 | 19144 | 26638 (end) | *S*. Typhimurium, *S*. Paratyphi B, *S*. Agona, *S*. Thompson, *S*. Weltevreden, *S*. Montevideo, *S*. Tennessee | 49.4 | FIG01048950: hypothetical protein, Predicted transcriptional regulator of the myo-inositol catabolic operon, Major myo-inositol transporter IolT, FIG01047800: hypothetical protein, Major myo-inositol transporter IolT, 5-deoxy-glucuronate isomerase (EC 5.3.1.-), Methylmalonate-semialdehyde dehydrogenase [inositol] (EC 1.2.1.27) |
| 28 | 80936 | 96603 (end) | *S*. Typhimurium, *S*. Paratyphi B, *S*. Agona, *S*. Thompson, *S*. Weltevreden, *S*. Montevideo, *S*. Tennessee | 50.4 | putative DNA-binding protein, Inosose dehydratase (EC 4.2.1.44), Myo-inositol 2-dehydrogenase 1 (EC 1.1.1.18), lysosomal glucosyl ceramidase-like protein, Inosose isomerase (EC 5.3.99.-), FIG01046005: hypothetical protein, 5-keto-2-deoxygluconokinase (EC 2.7.1.92) / uncharacterized domain, hypothetical protein, Epi-inositol hydrolase (EC 3.7.1.-), hypothetical protein, Myo-inositol 2-dehydrogenase 1 (EC 1.1.1.18), alpha-ketoglutarate permease, Inosose isomerase (EC 5.3.99.-),  Glyceraldehyde-3-phosphate ketol-isomerase (EC 5.3.1.1) |
| 28 | 72619 | 77082 | *S*. Typhimurium, *S*. Anatum, *S*. Heidelberg, *S*. Enteritidis | 56.7 | 2-dehydro-3-deoxyphosphogluconate aldolase (EC 4.1.2.14) in D-glucosaminate utilization operon, D-Glucosaminate-6-phosphate ammonia-lyase (EC 4.3.1.-), Metallo-dependent hydrolases, subgroup B, Putative inner membrane protein, Putative inner membrane protein |
| 28 (adjacent to tRNA-Leu-CAA | 31271 | 36266 | *S*. Typhimurium, *S*. Heidelberg, *S*. Anatum, *S*. Agona, *S*. Newport, *S*. Weltevreden | 51.4 | L-idonate, D-gluconate, 5-keto-D-gluconate transporter, 5-keto-D-gluconate 5-reductase (EC 1.1.1.69), L-idonate 5-dehydrogenase (EC 1.1.1.264), Gluconokinase (EC 2.7.1.12), Alcohol dehydrogenase (EC 1.1.1.1) |
| 28 (adjacent to tRNA-Leu-CAA | 9884 | 30096 | *S*. Typhimurium | 48.6 | Putative superfamily I DNA helicases, putative restriction endonuclease  putative ATP-dependent protease, putative cytoplasmic protein  putative cytoplasmic protein, putative ABC-type sugar/spermidine/putrescine transport system ATPase component, putative type II restriction enzyme methylase subunit, ATPase-like protein, putative cytoplasmic protein, putative inner membrane protein, hypothetical protein |
| 1 | 121920 | 131403 | *S*. Typhimurium, *S*. Heidelberg, *S*. Anatum, *S*. Thompson, *S*. Enteritidis | 52.5 | Methyl-accepting chemotaxis protein I (serine chemoreceptor protein), FIG01048313: hypothetical protein, Transcriptional regulatory protein levR, PTS system, mannose-specific IIA component (EC 2.7.1.69), PTS system, mannose-specific IIB component (EC 2.7.1.69), PTS system, mannose-specific IIC component (EC 2.7.1.69), PTS system, mannose-specific IID component (EC 2.7.1.69), Glucosamine--fructose-6-phosphate aminotransferase [isomerizing] (EC 2.6.1.16), Putative glucosamine-fructose-6-phosphate aminotransferase |
| 1 | 90174 | 95587 | *S*. Typhimurium, *S*. Heidelberg, *S*. Newport, *S*. Anatum, *S*. Thompson | 46.8 | Beta-fimbriae probable major subunit, Beta-fimbriae usher protein, Beta-fimbriae chaperone protein, Beta-fimbriae probable major subunit, Beta-fimbriae probable major subunit, hypothetical protein |
| 1 | 73732 | 77252 | *S*. Typhimurium, *S*. Anatum, *S*. Newport, *S*. Heidelberg, *S*. Agona, *S*. Weltevreden | 56.3 | Inner membrane protein CreD, Two-component response regulator CreC, Two-component response regulator CreB |
| 1 | 66382 | 73674 | *S*. Typhimurium, *S*. Newport, *S*. Anatum, *S*. Enteritidis | 46.7 | FIG00638667: hypothetical protein, putative inner membrane protein, putative fimbrial protein, Putative fimbrial chaperone protein, type 1 fimbriae anchoring protein FimD, putative fimbrial subunit, Putative major fimbrial subunit |
| 1 | 52817 | 53961 | *S*. Typhimurium, *S*. Heidelberg, *S*. Newport, *S*. Typhi, *S*. Anatum | 52.2 | FIG00638537: hypothetical protein, FIG00553873: hypothetical protein |
| 19 | 156172 | 160212 | *S*. Typhimurium, *S*. Newport, *S*. Anatum, *S*. Agona, *S*. Thompson, *S*. Weltevreden | 52.9 | FIG074102: hypothetical protein, Putative inner membrane protein, Putative inner membrane protein |
| 19 | 202415 | 206030 | *S*. Typhimurium, *S*. Anatum, *S*. Newport, *S*. Heidelberg, *S*. Enteritidis | 56.9 | Putative n-hydroxybenzoate hydroxylase, Maleylacetoacetate isomerase (EC 5.2.1.2) @ Glutathione S-transferase, zeta (EC 2.5.1.18), Fumarylacetoacetase (EC 3.7.1.2), Gentisate 1,2-dioxygenase (EC 1.13.11.4) |
| 21 (adjacent to tRNA-Lys-TTT) | 36142 | 40456 | *S*. Typhimurium, *S*. Heidelberg, *S*. Enteritidis | 47.8 | Putative exported protein precursor, Xanthosine phosphorylase (EC 2.4.2.1), Xanthosine permease, FIG00638837: hypothetical protein, Xanthosine operon regulatory protein XapR, LysR family |
| 21 | 163879 | 167737 | *S*. Typhimurium, *S*. Paratyphi A, *S*. Enteritidis | 54.8 | Polymyxin resistance protein PmrJ, predicted deacetylase, UDP-glucuronic acid oxidase (UDP-4-keto-hexauronic acid decarboxylating) (EC 1.1.1.305) / UDP-4-amino-4-deoxy-L-arabinose formyltransferase (EC 2.1.2.13), Polymyxin resistance protein ArnC, glycosyl transferase (EC 2.4.-.-) |
| 21 | 172635 | 176992 | *S*. Typhimurium, *S*. Heidelberg, *S*. Newport, *S*. Anatum, *S*. Enteritidis | 53.3 | Transcriptional regulator, IclR family, L-rhamnonate dehydratase (EC 4.2.1.90), L-rhamnonate transporter (predicted by genome context), 2,4-dihydroxyhept-2-ene-1,7-dioic acid aldolase (EC 4.1.2.-), Molybdopterin binding motif, CinA N-terminal domain / C-terminal domain of CinA type E |
| 21 | 192617 | 196093 | *S*. Typhimurium, *S*. Anatum, *S*. Thompson, *S*. Agona, *S*. Enteritidis | 40.7 | Transcriptional regulator, GntR family, Nitrate/nitrite transporter, Putative dehydratase protein STM2273 |
| 37 | 13527 | 17619 | *S*. Typhimurium, *S*. Anatum, *S*. Enteritidis | 54.6 | PTS system, fructose-specific IIB component (EC 2.7.1.69) / PTS system, fructose-specific IIC component (EC 2.7.1.69), Shikimate 5-dehydrogenase I gamma (EC 1.1.1.25), SgrR, sugar-phosphate stress, transcriptional activator of SgrS small RNA |
| 5 | 1 | 4377 | *S.* Typhimurium strain CFSAN001921, unnamed plasmid (CP006050.1) | 52.6 | hypothetical protein, tRNA pseudouridine synthase A( EC:4.2.1.70 ), hypothetical protein, hypothetical protein, hypothetical protein, mobilization protein MobC |
| 2 | 98164 (end) | 1 |  | 51.8 | FIG01199948: hypothetical protein, IncF plasmid conjugative transfer protein TraN, Mobile element protein, Mobile element protein, IncF plasmid conjugative transfer protein TraN, IncF plasmid conjugative transfer pilus assembly protein TraU, IncF plasmid conjugative transfer pilus assembly protein TraW, Conjugative signal peptidase TrhF, Conjugative transfer protein 345, IncF plasmid conjugative transfer pilus assembly protein TraC, Thiol:disulfide involved in conjugative transfer, Conjugative transfer protein 123, Conjugative transfer protein TraA, Conjugative transfer protein TraV, IncF plasmid conjugative transfer pilus assembly protein TraB, IncF plasmid conjugative transfer pilus assembly protein TraK, IncF plasmid conjugative transfer pilus assembly protein TraE, FIG01049278: hypothetical protein, FIG00291025: hypothetical protein, Conjugative transfer protein s043, Conjugative transfer protein 234, IncF plasmid conjugative transfer protein TraD, Conjugative transfer protein TraI, relaxase, hypothetical protein, DNA topoisomerase III (EC 5.99.1.2), hypothetical protein, hypothetical protein, hypothetical protein, hypothetical protein, hypothetical protein, hypothetical protein, hypothetical protein, FIG01049668: hypothetical protein, Chromosome (plasmid) partitioning protein ParB, Chromosome (plasmid) partitioning protein ParA, FIG01049723: hypothetical protein, Dihydropteroate synthase (EC 2.5.1.15), Phosphoglucosamine mutase (EC 5.4.2.10), Mobile element protein, Mobile element protein, Relaxase /helicase, Transcriptional regulator, TetR family, Tetracycline efflux protein TetA, Permease of the drug/metabolite transporter (DMT) superfamily, Amidases related to nicotinamidase, Mobile element protein, Mercuric resistance operon coregulatory, diguanylate cyclase/phosphodiesterase (GGDEF & EAL domains) with PAS/PAC sensor(s), Dihydrolipoamide dehydrogenase (EC 1.8.1.4), Mercuric transport protein, MerC, Periplasmic mercury(+2) binding protein, hypothetical protein, Mercuric transport protein, MerC, hypothetical protein, Periplasmic mercury(+2) binding protein, Mercuric resistance operon regulatory protein, RecD-like DNA helicase YrrC, hypothetical protein, hypothetical protein, hypothetical protein, putative DNA methyltransferase, hypothetical protein, Predicted transcriptional regulators, conserved hypothetical protein, hypothetical protein, hypothetical protein, hypothetical protein, hypothetical protein, FIG01206499: hypothetical protein, Cell division protein FtsH (EC 3.4.24.-), FIG01206602: hypothetical protein, FIG01049580: hypothetical protein, hypothetical protein, hypothetical protein, Transcriptional regulator, ArsR family, hypothetical protein, DNA-binding protein HU-beta, micrococcal nuclease (SNase-like), hypothetical protein, hypothetical protein, DNA primase (EC 2.7.7.-), hypothetical protein, conserved hypothetical protein, Periplasmic thiol:disulfide interchange protein DsbA, Periplasmic serine proteases (ClpP class), hypothetical protein, hypothetical protein, hypothetical protein, hypothetical protein, FIG01047316: hypothetical protein, plasmid replication protein RepA, hypothetical protein, hypothetical protein, Rod shape determination protein, hypothetical protein, Mobile element protein, Mobile element protein, Mobile element protein |
| 18 | 1 | 8484 (end) |  | 48.3 | FIG01202935: hypothetical protein, Recombination protein BET, Single-stranded DNA-binding protein, FIG01047344: hypothetical protein, Aerobic cobaltochelatase CobS subunit (EC 6.6.1.2), hypothetical protein  hypothetical protein, FIG01202318: hypothetical protein, Mobile element protein, hypothetical protein |
| 43 | 14400 (end) | 1 | *S*. Kentucky plasmid pCVM29188_146 | 47.2 | FIG00644632: hypothetical protein, FIG00643565: hypothetical protein, YadA, FIG00641434: hypothetical protein, YacB, Prevent host death protein, Phd antitoxin # D, FIG00643981: hypothetical protein, replication initiation protein, IncI1 plasmid conjugative transfer protein TraA, IncI1 plasmid conjugative transfer NusG-type transcription antiterminator TraB, IncI1 plasmid conjugative transfer protein TraC, IncI1 plasmid conjugative transfer protein PilI, IncI1 plasmid conjugative transfer protein PilJ, IncI1 plasmid conjugative transfer protein PilL, IncI1 plasmid conjugative transfer protein PilM |
| 49 | 1 | 29750 (end) |  | 52.8 | Beta-lactamase (EC 3.5.2.6), Outer membrane lipoprotein Blc, Quaternary ammonium compound-resistance protein SugE, hypothetical protein, FIG00641828: hypothetical protein, hypothetical protein  FIG00641804: hypothetical protein, 3',5'-cyclic-nucleotide phosphodiesterase (EC 3.1.4.17), FIG01046320: hypothetical protein, YdeA protein, YdfA protein, FIG01046273: hypothetical protein, hypothetical protein, FIG01048484: hypothetical protein, CcdA protein (antitoxin to CcdB), CcdB toxin protein, Resolvase, FIG01048970: hypothetical protein, ybiA, Putative stability/partitioning protein encoded within prophage CP-933T, stable plasmid inheritance protein, Error-prone, lesion bypass DNA polymerase V (UmuC), Error-prone repair protein UmuD, FIG00640646: hypothetical protein, FIG01048508: hypothetical protein, hypothetical protein, Adenine-specific methyltransferase (EC 2.1.1.72), putative cytoplasmic protein, YcgB, FIG00638373: hypothetical protein, Putative antirestriction protein, FIG00638373: hypothetical protein, FIG00638431: hypothetical protein, Single-stranded DNA-binding protein, FIG074102: hypothetical protein  putative plasmid stabilization protein, PsiB protein, PsiA protein, Transposase, Antirestriction protein ArdA, hypothetical protein |
| 55 | 13334 (end) | 1 |  | 51.1 | hypothetical protein, FIG01047460: hypothetical protein, TrbA, TrbB protein, FIG00640314: hypothetical protein, involved in conjugative DNA transfer, Nickel ABC transporter, periplasmic nickel-binding protein NikA (TC 3.A.1.5.3), FIG01047424: hypothetical protein, Z1226 protein, FIG01046993: hypothetical protein, FIG00642528: hypothetical protein, FIG01048117: hypothetical protein, Mobile element protein, YDFB protein, FIG01046738: hypothetical protein |
| 47 | 1 | 27743 (end) |  | 49.6 | FIG00641806: hypothetical protein, Phage minor tail protein, surface exclusion protein, IncI1 plasmid conjugative transfer integral membrane protein TraY, IncI1 plasmid conjugative transfer protein TraX, IncI1 plasmid conjugative transfer protein TraW, IncI1 plasmid conjugative transfer protein TraV, IncI1 plasmid conjugative transfer protein TraU, IncI1 plasmid conjugative transfer protein TraT, IncI1 plasmid conjugative transfer protein TraS, IncI1 plasmid conjugative transfer protein TraR, IncI1 plasmid conjugative transfer protein TraQ, IncI1 plasmid conjugative transfer protein TraP, IncI1 plasmid conjugative transfer protein TraO, IncI1 plasmid conjugative transfer protein TraN, IncI1 plasmid conjugative transfer protein TraM, IncI1 plasmid conjugative transfer protein TraL, IncI1 plasmid conjugative transfer DNA primase, Plasmid conjugative transfer endonuclease, IncI1 plasmid conjugative transfer protein TraJ, related to pilus biogenesis/retracton protein, IncI1 plasmid conjugative transfer protein TraI, IncI1 plasmid conjugative transfer protein TraH, IncI1 plasmid conjugative transfer protein TraG, IncI1 plasmid conjugative transfer protein TraF, IncI1 plasmid conjugative transfer protein TraE, Shufflon-specific DNA recombinase |
| 63 | 1 | 8916 (end) |  | 46.5 | IncI1 plasmid conjugative transfer prepilin PilS, IncI1 plasmid conjugative transfer inner membrane protein PilR, IncI1 plasmid conjugative transfer ATPase PilQ, IncI1 plasmid pilus assembly protein PilP, IncI1 plasmid pilus assembly protein PilO, IncI1 plasmid conjugative transfer lipoprotein PilN |
| 25 | 15188 | 27628 |  |  | hypothetical protein, Probable DNA recombinase, putative inner membrane protein, FIG074102: hypothetical protein, UDP-galactopyranose mutase (EC 5.4.99.9), Putative glycosyl transferase  Putative glycosyl transferase, O-antigen export system permease protein RfbD, O-antigen/lipopolysaccharide transport ATP-binding protein ABC transporter RfbE, putative glycosyltransferase, FIG01045205: hypothetical protein, Dolichol-phosphate mannosyltransferase (EC 2.4.1.83) in lipid-linked oligosaccharide synthesis cluster |
