# Supplementary material for: Genotypic and phenotypic characterization of multidrug resistant Salmonella Typhimurium and Salmonella Kentucky strains recovered from chicken carcasses
Source: PLoS One. 2017 May 8;12(5):e0176938. doi: 10.1371/journal.pone.0176938 (PMC5421757; doi:10.1371/journal.pone.0176938)
Supplement: S3 Table — (DOC) [file pone.0176938.s008.doc]

**S3 Table.** **Identification of gaps or genetic diferences in *Salmonella* Kentucky.**

| **Contig.** | **Start (bp)** | **End (bp)** | **Best Matches** | **% G+C** | **Function** |
| --- | --- | --- | --- | --- | --- |
| 11 (inserted adjacent to tRNA-Arg-TCT) | 277559 | 283176 | *S*, Bredeney CFSAN001080, *S*. Schwarzengrund CVM19633 | 44.6 | Mobile element protein, Mobile element protein, hypothetical protein, Polymyxin resistance protein ArnC, glycosyl transferase (EC 2.4.-.-), Bactoprenol-linked glucose translocase, copE1, Heavy metal sensor histidine kinase, Cobalt-zinc-cadmium resistance protein CzcA; Cation efflux system protein CusA |
| 11 | 293299 | 300797 | *S*. Typhimurium, *S*. Heidelberg, *S*. Anatum, *S*. Tennessee, *S*. Newport, *S*. Weltevreden | 54.1 | PTS system, mannose-specific IIA component (EC 2.7.1.69), PTS system, mannose-specific IIB component (EC 2.7.1.69), PTS system, mannose-specific IIC component (EC 2.7.1.69), PTS system, mannose-specific IID component (EC 2.7.1.69), Glucosamine--fructose-6-phosphate aminotransferase [isomerizing] (EC 2.6.1.16), Putative phosphosugar isomerase, NtrC family Transcriptional regulator, ATPase domain |
| 11 | 375951 | 380429 | *S*. Sloterdjik, *S*. Weltevreden, *S*. Tennessee, *S*. Agona, *S*. Anatum, *S*. Enteritidis | 51.3 | Hydrolase, UxaA family, Altronate hydrolase (EC 4.2.1.7), 2-keto-3-deoxygluconate permease (KDG permease), Propionate catabolism operon regulatory protein PrpR |
| 11 | 381034 | 389482 | *S*. Typhimurium, *S*. Enteritidis | 52.5 | putative cytoplasmic protein, Chaperone protein DnaK, FIG00638911: hypothetical protein, FIG00637867: hypothetical protein, Putative molecular chaperone, DnaJ family, FIG00637867: hypothetical protein, TETRATRICOPEPTIDE REPEAT FAMILY PROTEIN |
| 11 | 476433 | 481772 (end) | *S*. Typhimurium, *S*. Heidelberg, *S*. Anatum, *S*. Thompson, *S*. Enteritidis | 41.9 | L(+)-tartrate dehydratase alpha subunit (EC 4.2.1.32), Transcriptional regulator, lysR family, LysR-family transcriptional regulator STM0764, Putative membrane protein |
| 17 | 1 | 1093 | *S*. Typhimurium, *S*. Enteritidis | 60.7 | Membrane protein associated with oxaloacetate decarboxylase, Oxaloacetate decarboxylase beta chain (EC 4.1.1.3) |
| 17 | 52455 | 59005 (end) | *S.* Senftenberg*, S.* Newport*, S.* Tennessee | 51.7 | Chromosome initiation inhibitor, hypothetical protein, D-glucarate permease, 3-oxoacyl-[acyl-carrier protein] reductase (EC 1.1.1.100), Transketolase, N-terminal section (EC 2.2.1.1), Transketolase, C-terminal section (EC 2.2.1.1) |
| 8 | 1 | 19887 | *Enterobacter aerogenes* KCTC 2190 | 39.8 | FIG01046691: hypothetical protein, benzoate MFS transporter BenK, hypothetical protein, hypothetical protein, Porin B precursor, FIG01047079: hypothetical protein, hypothetical protein, Nucleoside permease NupG, hypothetical protein, Myo-inositol 2-dehydrogenase 1 (EC 1.1.1.18), Inosose isomerase (EC 5.3.99.-), Glucose-methanol-choline (GMC), oxidoreductase:NAD binding site, Glucokinase (EC 2.7.1.2), transcriptional regulator, Chromosome initiation inhibitor, Mobile element protein, Mobile element protein, FIG01048645: hypothetical protein, Mobile element protein |
| 3 | 483080 | 492306 | *S*. Sloterdjik, *S*. Weltevreden, *S*. Bareilly, *S*. Dublin, *S*. Enteritidis | 48.9 | Putative transport protein YdjK, MFS superfamily, Putative HTH-type transcriptional regulator YdjF, Hypothetical oxidoreductase YdjG (EC 1.-.-.-), Uncharacterized sugar kinase YdjH, Putative aldolase YdjI, Hypothetical zinc-type alcohol dehydrogenase-like protein YdjJ, Putative transport protein YdjK, MFS superfamily, Putative oxidoreductase YdjL, hypothetical protein, FIG01046088: hypothetical protein |
| 3 | 417334 | 426607 | *S*. Cubana, *S*. Weltevreden, *S*. Newport, *S*. Tennessee, *S*. Typhimurium, *S*. Anatum, *S*. Heidelberg | 50.6 | Coenzyme A transferase, Crotonobetainyl-CoA dehydrogenase (EC 1.3.99.-), Transcriptional regulator, AraC family, Electron transfer flavoprotein, beta subunit, Electron transfer flavoprotein, alpha subunit  Probable electron transfer flavoprotein-quinone oxidoreductase FixC (EC 1.5.5.-), Ferredoxin-like protein FixX, Long-chain-fatty-acid--CoA ligase (EC 6.2.1.3) |
| 3 | 412133 | 416376 | *S*. Tennessee, *S*. Typhimurium, *S*. Newport, *S*. Enteritidis | 45.4 | Putative cytoplasmic protein, Putative transport system permease protein, Putative transport system permease protein, Shikimate/quinate 5-dehydrogenase I beta (EC 1.1.1.282) |
| 3 | 237550 | 240824 | *S*. Senftenberg, *S*. Agona, *S*. Newport, *S*. Cubana, *S*. Enteritidis | 46 | L-galactonate dehydrogenase (EC 1.1.1.-) / Sorbitol dehydrogenase (EC 1.1.1.14), Hexuronate utilization operon transcriptional repressor ExuR, Choloylglycine hydrolase (EC 3.5.1.24), hypothetical protein |
| 3 | 216505 | 234486 | *S*. Senftenberg, *S*. Agona, *S*. Typhimurium, *S*. Tennessee | 44.2 | putative coiled-coil protein, putative dehydrogenase, Transcriptional regulator, LysR family, FIG01045672: hypothetical protein, TolA protein,  hypothetical protein, hypothetical protein, FIG01048272: hypothetical protein, FIG01046001: hypothetical protein, RelB/StbD replicon stabilization protein (antitoxin to RelE/StbE), RelE/StbE replicon stabilization toxin, RidA/YER057c/UK114 superfamily, group 7, YjgH-like protein, S-adenosylmethionine:tRNA ribosyltransferase-isomerase (EC 5.-.-.-), Transcriptional regulator, MarR family, Oxygenase-like protein  12-TMS multidrug efflux protein homolog |
| 3 | 191593 | 199483 | *S*. Typhimurium, *S*. Newport, *S*. Thomopson, *S*. Weltevreden, *S*. Enteritidis | 50.9 | Gfa-like protein, Outer membrane porin protein NmpC precursor, Permease of the drug/metabolite transporter (DMT) superfamily, Formate dehydrogenase N alpha subunit (EC 1.2.1.2) @ selenocysteine-containing, Formate dehydrogenase N beta subunit (EC 1.2.1.2), Formate dehydrogenase N gamma subunit (EC 1.2.1.2) |
| 3 | 175974 | 189020 | *S*. Senftenberg, *S*. Agona, *S*. Weltevreden, *S*. Typhimurium, *S*. Enteritidis | 53.1 | Putative outer membrane lipoprotein, L-asparagine permease, hypothetical protein, FIG074102: hypothetical protein, hypothetical protein, N-hydroxyarylamine O-acetyltransferase (EC 2.3.1.118), Phenazine biosynthesis protein PhzF, Respiratory nitrate reductase gamma chain (EC 1.7.99.4), Respiratory nitrate reductase delta chain (EC 1.7.99.4), Respiratory nitrate reductase beta chain (EC 1.7.99.4), Respiratory nitrate reductase alpha chain (EC 1.7.99.4), Nitrate/nitrite transporter NarK |
| 3 | 138758 | 145466 | *S*. Tennessee, *S*. Typhimurium, *S*. Paratyphi B, *S*. Anatum, *S*. Newport, *S*. Heidelberg, *S*. Thompson | 51.3 | FIG01200701: possible membrane protein, Lactate 2-monooxygenase (EC 1.13.12.4), Lactate 2-monooxygenase (EC 1.13.12.4), Aminoglycoside 6'-N-acetyltransferase, Putative arylsulfatase regulatory protein, Ribulose-phosphate 3-epimerase (EC 5.1.3.1), PTS system, IIA component, Putative nucleoside triphosphatase, PTS system, galactitol-specific IIC component (EC 2.7.1.69), Putative phosphotransferase enzyme |
| 3 | 127516 | 135121 | *S*. Senftenberg, *S*. Agona, *S*.  Weltevreden, *S*. Cubana, *S*. Enteritidis | 50.1 | FIG01045328: hypothetical protein, FIG01045328: hypothetical protein  hypothetical protein, FrmR: Negative transcriptional regulator of formaldehyde detoxification operon, S-(hydroxymethyl)glutathione dehydrogenase (EC 1.1.1.284), Methyl-accepting chemotaxis protein III (ribose and galactose chemoreceptor protein), LysR family transcriptional regulator YdcI, FIG074102: hypothetical protein |
| 3 | 119966 | 125746 | *S*. Typhi, *S*. Agona, *S*. Anatum, *S*. Anatum, *S*. Typhimurium, *S*. Heidelberg, *S*. Newport | 41.8 | putative membrane protein, putative amino acid ABC transporter, ABC transporter ATP-binding subunit, Putative ABC amino acid transporter permease, Putative periplasmic binding protein, Putative inner membrane protein |
| 3 | 97729 | 101152 | *S*. Agona, *S*. Cubana, *S*. Tennessee | 39.3 | FIG01046091: hypothetical protein, FIG01046091: hypothetical protein |
| 3 | 72482 | 77052 | *S*. Tennessee, *S*. Agona, *S*. Anatum, *S*. Enteritidis | 52 | FIG00553873: hypothetical protein, LysR family transcriptional regulator YcjZ, FIG01047110: hypothetical protein, oxidoreductase, aldo/keto reductase family, Uncharacterized oxidoreductase YjgI (EC 1.-.-.-),  Putative HTH-type transcriptional regulator YjgJ, TetR family |
| 2 | 1 | 30,058 | *S*. Newport, *S*. Weltevreden, *S*. Enteritidis | 47.6 | FIG01046332: hypothetical protein, hypothetical bacteriophage protein, FIG01046332: hypothetical protein, hypothetical protein, hypothetical protein, FIG00640812: hypothetical protein, Phage tail fiber protein, unknown protein encoded by bacteriophage BP-933W, FIG00640276: hypothetical protein, FIG00642416: hypothetical protein, Phage protein,  FIG01047504: hypothetical protein, Phage protein, secreted effector protein, hypothetical protein, Phage tail fibers, hypothetical protein, FIG01048391: hypothetical protein, hypothetical protein, Error-prone repair protein UmuC, Mobile element protein, hypothetical protein, FIG036507: Fimbriae usher protein StdB, hypothetical protein, hypothetical protein |
| 22 | 245,459 | 278,808 (end) | *S*. Typhimurium, *S*. Anatum | 51.2 | FIG01046976: hypothetical protein, Phage protein, hypothetical protein, FIG01047003: hypothetical protein, FIG01048296: hypothetical protein, Gifsy-2 prophage RecT, Exodeoxyribonuclease VIII (EC 3.1.11.-), FIG01048001: hypothetical protein, FIG01045596: hypothetical protein, hypothetical protein, putative regulator, FIG01047021: hypothetical protein, Primosomal protein I, FIG01046796: hypothetical protein, FIG01049183: hypothetical protein, MokW protein, FIG01047714: hypothetical protein, hypothetical bacteriophage protein, FIG01049733: hypothetical protein, Phage antitermination protein Q, FIG01048836: hypothetical protein, hypothetical protein, Phage tail fibers, phage lysozyme( EC:3.2.1.17 ), Putative endopeptidase, hypothetical protein, FIG01047729: hypothetical protein, Phage terminase, small subunit, FIG01045251: hypothetical protein, Phage protein, FIG00643399: hypothetical protein, conserved phage protein, FIG00641463: hypothetical protein, FIG00638618: hypothetical protein, Phage protein, FIG00642812: hypothetical protein, Phage tail fiber protein, Mobile element protein, Mobile element protein |
| 22 (adjacent to tRNA-Asn-GTT) | 110347 | 114782 | *S*. Tennessee, *S*. Anatum, *S*. Newport, *S*. Agona, *S*. Thompson, *S*. Montevideo | 55.8 | Arsenical resistance operon repressor, Arsenate reductase, Arsenical resistance operon trans-acting repressor ArsD, Arsenical pump-driving ATPase, Arsenical-resistance protein ACR3, Hypothetical protein, |
| 13 | 22555 | 38107 | *S*. Senftenberg, *S*. Tennessee, *S*.  Thompson | 41.8 | FIG074102: hypothetical protein, Putative inner membrane protein, Putative inner membrane protein, FIG01200701: possible membrane protein, FIG01200701: possible membrane protein, hypothetical protein, hypothetical protein, hypothetical protein, hypothetical protein, core protein, hypothetical protein, hypothetical protein, core protein, hypothetical protein, hypothetical protein, FIG01200701: possible membrane protein |
| 13 | 81286 | 84901 | *S*. Weltevreden, *S*. Agona, *S*. Typhimurium, *S*. Anatum | 56.8 | Putative n-hydroxybenzoate hydroxylase, Maleylacetoacetate isomerase (EC 5.2.1.2) @ Glutathione S-transferase, zeta (EC 2.5.1.18), Fumarylacetoacetase (EC 3.7.1.2), Gentisate 1,2-dioxygenase (EC 1.13.11.4) |
| 16 | 381515 | 384991 | *S*. Sloterdjik, *S*. Weltevreden, *S*. Paratyphi A, *S*. Typhimurium, *S*. Anatum, *S*. Enteritidis | 40.7 | putative MR-MLE-family protein, Nitrate/nitrite transporter, Transcriptional regulator, GntR family |
| 16 | 361409 | 365766 | *S*. Cubana, *S*. Tennessee, *S*. Montevideo, *S*. Agona, *S*. Typhi, *S*. Anatum, *S*. Thompson | 53.4 | Molybdopterin binding motif, CinA N-terminal domain / C-terminal domain of CinA type E, 2-dehydro-3-deoxy-L-rhamnonate aldolase (EC 4.1.2.n3), L-rhamnonate transporter (predicted by genome context), L-rhamnonate dehydratase (EC 4.2.1.90), Transcriptional regulator, IclR family |
| 16 | 352652 | 356510 | *S*. Sloterdjik, *S*. Heidelberg, *S*. Enteritidis | 54.7 | Polymyxin resistance protein ArnC, glycosyl transferase (EC 2.4.-.-), UDP-glucuronic acid oxidase (UDP-4-keto-hexauronic acid decarboxylating) (EC 1.1.1.305) / UDP-4-amino-4-deoxy-L-arabinose formyltransferase (EC 2.1.2.13), Polymyxin resistance protein PmrJ, predicted deacetylase |
| 16 | 328844 | 339938 | *S*. Typhimurium, *S*. Cubana, *S*. Agona, *S*. Senftenberg, *S*. Tennessee, *S*. Sloterdjik | 58.6 | FIG01046494: hypothetical protein, hypothetical protein, FIG01046512: hypothetical protein, AIDA autotransporter-like protein |
| 16 (adjacent to tRNA-Lys-TTT) | 216587 | 220901 | *S*. Agona, *S*. Typhi, *S*. Enteritidis | 47.7 | Xanthosine operon regulatory protein XapR, LysR family, FIG00638837: hypothetical protein, Xanthosine permease, Xanthosine phosphorylase (EC 2.4.2.1), Putative exported protein precursor |
| 16 | 118041 | 130782 | *S*. Senftenberg, *S*. Typhimurium | 44.4 | Putative outer membrane lipoprotein, Putative membrane protein, hypothetical protein, Mobile element protein, FIG01047222: hypothetical protein, FIG074102: hypothetical protein, Mobile element protein, Inner membrane protein, VapB protein (antitoxin to VapC), Mobile element protein, Mobile element protein, Mobile element protein, Mobile element protein, putative integrase, hypothetical protein, Plasmid-related protein, hypothetical protein, phage-related integrase |
| 16 | 26922 | 30099 | *S*. Typhimurium, *S*. Heidelberg, *S*. Typhi, *S*. Paratyphi A, *S*. Anatum, *S*. Newport | 45.3 | 2-dehydropantoate 2-reductase (EC 1.1.1.169), Putative transmembrane transport protein, Transcriptional regulator, LysR family |
| 4 | 303233 | 318628 | *E. coli* FHI98 | 44.8 | prophage CP4-57 integrase, hypothetical protein, Mobile element protein  Mobile element protein, hypothetical protein, DNA helicase, putative  NgrB, Antirestriction protein klcA, DNA repair protein RadC, Uncharacterized protein YkfH, YeeU protein (antitoxin to YeeV), YpjF toxin protein, Flagellar synthesis: repressor of fliC, Flagellar biosynthesis protein FliC |
| 4 | 166630 | 171721 | *S*. Senftenberg, *S*. Thompson, *S*.  Anatum, *S*. Agona, *S*. Paratyphi B, *S*. Enteritidis | 45.2 | putative cytoplasmic protein, FIG032766: hypothetical protein, FIG001353: Acetyltransferase, transposase, transposase, Serine/threonine specific protein phosphatase 2 (EC 3.1.3.16), FIG074102: hypothetical protein, FIG01046482: hypothetical protein, hypothetical protein |
| 4 | 154684 | 160762 | *S*. Weltevreden, *S*. Typhimurium, *S*. Enteritidis | 58.3 | Gluconate permease  Nucleoside-diphosphate-sugar epimerases  Hydroxypyruvate isomerase (EC 5.3.1.22)  Ribulose-5-phosphate 4-epimerase and related epimerases and aldolases  FIG00641944: hypothetical protein  D-beta-hydroxybutyrate dehydrogenase (EC 1.1.1.30) |
| 4 | 107455 | 115818 | *S*. Senftenberg, *S*. Typhimurium, *S*. Agona, *S*. Bareilly, *S*. Cubana | 49.5 | FIG01047509: hypothetical protein, Fimbriae usher protein StfC, Periplasmic fimbrial chaperone, Minor fimbrial subunit StfE, MrfF, Fimbrial subunit, FIG01046092: hypothetical protein, FIG027937: secreted protein, Transcriptional regulator, ArsR family |
| 4 | 15420 | 24208 | *S*. Cubana, *S*. Typhimurium, *S*. Enteritidis | 50 | FIG01045819: hypothetical protein, Putative outer membrane protein, Probable fimbrial chaperone protein, FIG036507: Fimbriae usher protein StdB, Putative fimbrial-like protein, FIG00638667: hypothetical protein, Attachment invasion locus protein precursor, hypothetical protein, FIG01047567: hypothetical protein, hypothetical protein |
| 12 | 177313 | 182865 | *S*. Newport, *S*. Weltevreden, *S*. arizonae, *S*. diarizonae, *S*. bongori | 50.4 | PTS system, IIA component, PTS system, mannitol-specific cryptic IIB component (EC 2.7.1.69) / PTS system, mannitol-specific cryptic IIC component (EC 2.7.1.69), Putative oxidoreductase linked to yggC, Fructose-1,6-bisphosphatase, GlpX type (EC 3.1.3.11), Putative transcriptional regulator, Uridine kinase family protein |
| 12 | 164899 | 172747 | *S*. Cubana, *S*. Agona, *S*. Heidelberg, *S*. Anatum, *S*. Thompson, *S*. Newport, *S*. Enteritidis | 48.1 | Putative outer membrane lipoprotein, Hexuronate utilization operon transcriptional repressor ExuR, hypothetical protein, D-mannonate oxidoreductase (EC 1.1.1.57), Sorbitol dehydrogenase (EC 1.1.1.14), (R)-2-hydroxyacid dehydrogenase, similar to L-sulfolactate dehydrogenase (EC 1.1.1.272), Putative mannitol dehydrogenase, hypothetical protein, FIG01048344: hypothetical protein |
| 12 (inserted at tRNA-Phe-GAA) | 130241 | 137297 | *S*. Tennessee, *S*. Senftenberg, *S*. Cubana, *S*. Typhi, *S*. Paratyphi, *S*. Agona | 38 | hypothetical protein, possible membrane protein, FIG01045941: hypothetical protein, FIG01200701: possible membrane protein, hypothetical protein, bacteriocin immunity protein, bacteriocin immunity protein, hypothetical protein, hypothetical protein, hypothetical protein, bacteriophage integrase, FIG01047955: hypothetical protein, bacteriophage integrase |
| 25 | 8169 | 12296 | *S*. Senftenberg, *S*. Anatum, *S*.  Agona, *S*. Newport, *S*. Typhimurium, *S*. Thompson, *S*. Enteritidis | 53.2 | PTS system, galactitol-specific IIA component (EC 2.7.1.69), PTS system, galactitol-specific IIB component (EC 2.7.1.69), PTS system, galactitol-specific IIC component (EC 2.7.1.69), Galactitol-1-phosphate 5-dehydrogenase (EC 1.1.1.251), Galactitol utilization operon repressor |
| 25 | 97067 | 105634 | *S*. Tennessee, *S*. Anatum, *S*.  Thompson, *S*. Weltevreden, *S*. Typhimurium, *S*. Enteritidis | 55.7 | Membrane protein associated with oxaloacetate decarboxylase, Oxaloacetate decarboxylase beta chain (EC 4.1.1.3), Oxaloacetate decarboxylase beta chain (EC 4.1.1.3), Oxaloacetate decarboxylase alpha chain (EC 4.1.1.3), Oxaloacetate decarboxylase gamma chain (EC 4.1.1.3), L(+)-tartrate dehydratase beta subunit (EC 4.2.1.32), L(+)-tartrate dehydratase alpha subunit (EC 4.2.1.32), Putative membrane protein, Transcriptional regulator, GntR family, Transcriptional regulator, GntR family |
| 25 | 139110 | 144730 | *S*. Tennessee, *S*. Agona, *S*. Enteritidis | 49.2 | Transcription repressor of multidrug efflux pump acrAB operon, TetR (AcrR) family, RND efflux system, membrane fusion protein CmeA, RND efflux system, inner membrane transporter CmeB, FIG01045506: hypothetical protein |
| 14 | 59802 | 68251 | *S*. Tennessee, *S*. Agona, *S*. Anatum, *S*. Enteritidis | 51.4 | Putative inner membrane protein, Phosphate ABC transporter, periplasmic phosphate-binding protein PstS (TC 3.A.1.7.1), Glycerol dehydrogenase (EC 1.1.1.6), Nitrate/nitrite transporter, Dihydroxy-acid dehydratase (EC 4.2.1.9), 4-hydroxy-tetrahydrodipicolinate synthase (EC 4.3.3.7), Transcriptional regulator, ArsR family |
| 14 | 85135 | 89935 | *S*. Tennessee, *S*. Anatum, *S*. Typhimurium, *S*. Heidelberg, *S*. Enteritidis | 46.8 | Putative acetyltransferase, hypothetical protein, Putative ribokinase  FIG074102: hypothetical protein, Putative inner membrane protein, Puative phophotriesterase |
| 14 | 121097 | 126249 | *S*. Tennessee, *S*. Heidelberg, *S*. Newport, *S*. Typhimurium, *S*. Enteritidis | 57.6 | FIG01045128: hypothetical protein, ABC-type multidrug transport system, permease component, Putative membrane protein, hypothetical protein |
| 14 | 135412 | 136786 | *S*. Typhimurium, *S*. Agona, *S*.  Newport, *S*. Paratyphi A, *S*. Typhi, *S*. Weltevreden | 44.7 | FIG00545237: hypothetical protein, Putative membrane protein |
| 14 (adjacent to tRNA-Pro-CGG) | 185848 | 189512 | *S*. Typhimurium, *S*. Enteritidis | 56.7 | Xanthine permease, Putative PQQ enzyme repeat, Putative lacI-family transcriptional regulator |
| 14 | 191901 | 197374 | *S*. Tennessee, *S*. Cubana, *S*. Agona, *S*. Senftenberg, *S*. Typhimurium | 51.2 | putative fimbrial protein precursor, Putative fimbrial protein, type 1 fimbriae anchoring protein FimD, Chaperone protein lpfB precursor, Long polar fimbria protein A precursor |
| 14 | 222684 | 234888 | *S*. Bredeney, *S*. Schwarzengrund, *S*.  Anatum, *S*. Typhimurium, *S*. Agona, *S*. Enteritidis | 54.5 | 3-dehydro-L-gulonate 2-dehydrogenase (EC 1.1.1.130), Putative sugar isomerase involved in processing of exogenous sialic acid, Putative chemotaxis protein, resembles cheA, 2,3-diketo-L-gulonate TRAP transporter small permease protein yiaM, 2,3-diketo-L-gulonate TRAP transporter large permease protein yiaN, 2,3-diketo-L-gulonate-binding periplasmic protein yiaO precursor, L-xylulose/3-keto-L-gulonate kinase (EC 2.7.1.-), 3-keto-L-gulonate 6-phosphate decarboxylase homolog, L-xylulose 5-phosphate 3-epimerase (EC 5.1.3.-) homolog, L-ribulose-5-phosphate 4-epimerase (EC 5.1.3.4), Putative arylsulfatase regulatory protein, Putative glycosyl hydrolase of unknown function (DUF1680) |
| 14 (adjacent to tRNA-SeC(p)-TCA | 315799 | 323621 | *S*. Paratyphi A, *S*. Typhi, *S*.  Agona, *S*. Newport, *S*. Montevideo, *S*. Aantum | 45.9 | hypothetical protein, RmbA, autotransporter, YqeJ protein, Putative sensory transducer, hypothetical protein, Nicotinamidase family protein YcaC |
| 14 | 329738 | 349509 | *S*. Senftenberg, *S*. *S*. Thompson, *S*. Newport | 50.9 | Uncharacterized protein YfaD, 4-hydroxy-2-oxoglutarate aldolase (EC 4.1.3.16), D-Glucosaminate-6-phosphate ammonia-lyase (EC 4.3.1.-), PTS system, mannose-specific IID component (EC 2.7.1.69), PTS system, mannose-specific IIC component, PTS system, gluconate-specific IIB component (EC 2.7.1.69), PTS system, mannose-specific IIA component, Transcriptional regulatory protein zraR, Putative secreted protein, hypothetical protein, Beta-glucosidase (EC 3.2.1.21), Putative transport protein, FIG074102: hypothetical protein, Putative DNA-binding protein in cluster with Type I restriction-modification system, FIG01046949: hypothetical protein, hypothetical protein, Phosphotransferase system HPr enzyme STM3779, Fructose-bisphosphate aldolase (EC 4.1.2.13), Putative carbohydrate kinase in cluster with fructose-bisphosphate aldolase, Putative carbohydrate PTS system, IIC component (EC 2.7.1.69), Putative carbohydrate PTS system, IIB component (EC 2.7.1.69), Putative carbohydrate PTS system, IIA component (EC 2.7.1.69), Putative transcriptional regulator of unknown carbohydrate utilization cluster, GntR family |
| 10 | 11037 | 14434 | *S*. Senftenberg, *S*.  Agona, *S*. Typhi, *S*. *bongori* | 54.8 | corresponds to STY3950 from Accession AL513382: Salmonella typhi CT18, Outer membrane protein/protective antigen OMA87, FIG01046532: hypothetical protein, hypothetical protein |
| 10 | 40756 | 44848 | *S*. Cubana, *S*. Anatum, *S*. Agona, *S*. Typhimurium, *S*. Weltevreden, *S*. Newport, *S*. Heidelberg | 54.7 | PTS system, fructose-specific IIB component (EC 2.7.1.69) / PTS system, fructose-specific IIC component (EC 2.7.1.69), Shikimate 5-dehydrogenase I gamma (EC 1.1.1.25), SgrR, sugar-phosphate stress, transcriptional activator of SgrS small RNA |
| 26 | 45800 | 47026 | *S*. Senftenberg, *S*. Cubana, *S*.  Agona, *S*. Anatum, *S*. Weltevreden, *S*. Typhimurium, *S*. Typhi | 41.2 | hypothetical protein, Putative inner membrane protein, Putative inner membrane protein |
| 19 | 18005 | 22696 | *S*. Agona, *S*. Thompson, *S*. Weltevreden, *S*. Typhimurium, *S*. Enteritidis | 48.3 | Putative hydrolase, FIG01046022: hypothetical protein, Hypothetical radical SAM family enzyme in interesting gene cluster, FIG01124638: hypothetical protein, FIG00638667: hypothetical protein |
| 19 | 47850 | 55835 | *S*. Agona, *S*. Typhimurium | 47.8 | Putative lipoprotein, FIG01045194: hypothetical protein, FIG01048471: hypothetical protein, FIG01047325: hypothetical protein, FIG01047737: hypothetical protein, FIG00638858: hypothetical protein |
| 19 | 80341 | 94908 | *S*. Typhimurium, *S*. Heidelberg, *S*. Agona, *S*. Enteritidis | 51.6 | Melibiose carrier protein, Na+/melibiose symporter, Fructokinase (EC 2.7.1.4), ADP-ribosylglycohydrolase YegU (EC 3.2.-.-), Transcriptional regulator, GntR family, FIG00638667: hypothetical protein, Putative periplasmic protein, Mannose-6-phosphate isomerase (EC 5.3.1.8), Autoinducer 2 (AI-2) kinase LsrK (EC 2.7.1.-), LsrR, transcriptional repressor of lsr operon, Autoinducer 2 (AI-2) ABC transport system, fused , AI2 transporter subunits and ATP-binding component, Autoinducer 2 (AI-2) ABC transport system, membrane channel protein LsrC, Autoinducer 2 (AI-2) ABC transport system, membrane channel protein LsrD, Autoinducer 2 (AI-2) ABC transport system, periplasmic AI-2 binding protein LsrB, Autoinducer 2 (AI-2) aldolase LsrF (EC 4.2.1.-), Autoinducer 2 (AI-2) modifying protein LsrG, Ribulose-phosphate 3-epimerase (EC 5.1.3.1) |
| 19 | 110759 | 113458 | *S*. Paratyphi B, *S*. Anatum, *S*. Heidelberg, *S*. Newport, *S*. Typhimurium, *S*. Enteritidis | 47.5 | putative outer membrane lipoprotein, FIG01046148: hypothetical protein, Putative arylsulfate sulfotransferase (EC 2.8.2.22), FIG01045707: hypothetical protein |
| 28 | 17772 | 19808 | *S*. Choleraesuis B, *S*. Weltevreden, *S*. Enteritidis | 32.3 | STMF1.14 protein, FIG01046757: hypothetical protein, FIG01046757: hypothetical protein, putative cytoplasmic protein |
| 18 | 17301 | 19232 | *S*. Paratyphi C, *S*. Agona, *S*. Typhimurium, *S*. Heidelberg, *S*. Enteritidis | 48.1 | Sodium-dependent transporter, FIG01046261: hypothetical protein, putative cytoplasmic protein, hypothetical protein, putative cytoplasmic protein |
| 18 | 19685 | 38348 | *S*. Agona, *S*. Tennessee, *S*. Typhimurium | 51.3 | putative phage tail fiber protein H, Putative phage tail protein, Phage baseplate, putative bacteriophage baseplate protein, hypothetical protein, putative inner membrane protein, Polymyxin resistance protein ArnC, glycosyl transferase (EC 2.4.-.-), putative phage glucose translocase, hypothetical protein, Putative phage baseplate component, Gene D protein,  Putative inner membrane protein, FIG00639790: hypothetical protein, Phage tail length tape-measure protein, hypothetical protein, FIG00639134: hypothetical protein, Putative phage tail core protein, Phage tail sheath monomer, FIG074102: hypothetical protein, FIG01045878: hypothetical protein, Putative inner membrane protein, Phage lysine, Putative inner membrane protein, putative cytoplasmic protein |
| 7 | 322652 | 333936 | *S*. Bareilly, *S*. Anatum | 55.5 | ISSod13, transposase, FIG01045493: hypothetical protein, hypothetical protein, hypothetical protein, major pilu subunit operon regulatory protein PapB, FIG01045656: hypothetical protein, PefC, Putative fimbrial chaperone protein, FIG01046636: hypothetical protein, FIG01046857: hypothetical protein, K88 minor fimbrial subunit faeH precursor, K88 minor fimbrial subunit faeI precursor, FIG01047379: hypothetical protein, Probable regulatory protein, Outer membrane protein assembly factor YaeT precursor |
| 7 | 221441 | 225904 | *S*. Agona, *S*. Tennessee, *S*. Newport, *S*. Heidelberg, *S*. Typhimurium, *S*. Enteritidis | 56.8 | Putative inner membrane protein, Putative inner membrane protein, Metallo-dependent hydrolases, subgroup B, D-Glucosaminate-6-phosphate ammonia-lyase (EC 4.3.1.-), 2-dehydro-3-deoxyphosphogluconate aldolase (EC 4.1.2.14) in D-glucosaminate utilization operon |
| 7 (adjacent to tRNA-Leu-CAA) | 162759 | 179791 | unique | 38.2 | Integrase, hypothetical protein, hypothetical protein, Transcriptional regulator in PFGI-1-like cluster, FIG00733113: hypothetical protein, hypothetical protein, hypothetical protein, DNA sulfur modification protein DndD, 3'-phosphoadenosine 5'-phosphosulfate sulfurtransferase DndC, Cysteine desulfurase (EC 2.8.1.7), IscS subfamily, Mrr restriction system protein, Mobile element protein, FIG01048645: hypothetical protein, Mobile element protein |
| 7 | 157980 | 162246 | *S*. Paratyphi B, *S*. Tennessee, *S*. Cubana, *S*. Enteritidis | 47.6 | FIG007491: hypothetical protein YeeN, 3-demethylubiquinone-9 3-methyltransferase (EC 2.1.1.64), FIG01045803: hypothetical protein, FIG01046166: hypothetical protein, FIG074102: hypothetical protein |
| 7 | 129203 | 139367 | *S*. Paratyphi B, *S*. Abony | 50.4 | Putative inner membrane protein, FIG00638399: hypothetical protein, Type I restriction-modification system, specificity subunit S (EC 3.1.21.3), Type I restriction-modification system, DNA-methyltransferase subunit M (EC 2.1.1.72), Type I restriction-modification system, restriction subunit R (EC 3.1.21.3), Mrr restriction system protein, FIG01045296: hypothetical protein, FIG00638610: hypothetical protein |
| 7 | 84376 | 89578 | *S*. Senftenberg, *S*. Agona, *S*. Thompson, *S*. Typhimurium, *S*. Heidleberg, *S*. Newport, *S*. Anatum | 47.7 | Beta-fimbriae probable major subunit, Beta-fimbriae usher protein, Beta-fimbriae chaperone protein, Beta-fimbriae probable major subunit, Beta-fimbriae probable major subunit |
| 7 | 67725 | 71245 | *S*. Cubana *S*. Anatum, *S*. Newport, *S*. Typhimurium, *S*. Heidleberg, *S*. Paratyphi A | 56 | Two-component response regulator CreB, Two-component response regulator CreC, Inner membrane protein CreD |
| 9 | 102269 | 113162 | *S*. Senftenberg *S*. Agona, *S*. Heidelberg, *S*. Thompson | 51.6 | FIG01047318: hypothetical protein, FIG01046572: hypothetical protein,  FIG01046124: hypothetical protein, FIG01046819: hypothetical protein,  FIG01046410: hypothetical protein, hypothetical protein, FIG01045138: hypothetical protein, cellular communication/signal transduction, FIG01047211: hypothetical protein, FIG01045153: hypothetical protein, 2-keto-3-deoxygluconate permease (KDG permease), hypothetical protein, 4-hydroxythreonine-4-phosphate dehydrogenase (EC 1.1.1.262), DeoR family transcriptional regulator probably related to glycerate, glycolaldehyde metabolism |
| 9 | 78430 | 85427 | *S*. Senftenberg, *S*. Tennessee, *S*. Thompson, *S*. Paratyphi A, *S*. Heidelberg | 49.4 | Fimbrial protein Yad-like, Fimbrial protein YadK, Fimbrial protein YadL, Fimbrial protein YadM-like, Outer membrane usher protein HtrE, Chaperone protein EcpD, Fimbrial protein YadN-like |
| 9 | 52022 | 59221 | *S*. Senftenberg, *S*. Anatum, *S*.  Enteritidis | 50.9 | hypothetical protein, hypothetical protein, Major fimbrial subunit StfA, Fimbriae usher protein StfC, Periplasmic fimbrial chaperone StfD, Minor fimbrial subunit StfE, Minor fimbrial subunit StfF, Minor fimbrial subunit StfG, Uncharacterized protein YadU in stf fimbrial cluster |
| 11, adjacent to T6SS | 6692 | 13447 | *S*. Typhimurium, Paratyphi C, Choleraesuis, Cubana | 49 | LysR-family transcriptional regulator SinR, hypothetical protein, Polysaccharide deacetylase, Putative fimbrial structural subunit, FIG034929: Fimbriae usher protein SafC, Periplasmic fimbrial chaperone protein (*safABCD*) |
| 11 (inserted adjacent to tRNA-Thr-CGT) | 33160 | 41366 | *S*. Typhimurium | 49.4 | Mobile element protein, glycerol dehyrdratase activator, Glycerol dehydratase reactivation factor large subunit, FIG074102: hypothetical protein, putative permease, 3-isopropylmalate dehydratase large subunit (EC 4.2.1.33), 3-isopropylmalate dehydratase small subunit (EC 4.2.1.33), Fumarylacetoacetate hydrolase family protein, Putative hydrolase or acyltransferase, Putative LysR family transcriptional regulator |
| 11 (includes γ_4_ fimbriae *stbABCDE*) | 42478 | 65067 | *S*. Tennessee, *S*. Agona, *S*. Montevideo, *S*. Weltevreden, *S*. Enteritidis | 50.5 | Probable secreted protein, Putative pilus chaperone, PapD family, Putative exported protein precursor, outer membrane fimbrial usher protein, Putative fimbrial chaperone, Fimbrial protein precursor, Possible transmembrane regulator, Putative inner membrane protein, diguanylate cyclase/phosphodiesterase (GGDEF & EAL domains) with PAS/PAC sensor(s), Possible transmembrane regulator, Putative inner membrane protein, hypothetical protein, Attachment invasion locus protein precursor, Possible transcriptionl regulator, Probable secreted protein, Putative outer membrane lipoprotein, RND efflux system, outer membrane lipoprotein CmeC, RND efflux system, inner membrane transporter CmeB, Multidrug efflux membrane fusion protein MexE, hypothetical protein, Lead, cadmium, zinc and mercury transporting ATPase (EC 3.6.3.3) (EC 3.6.3.5); Copper-translocating P-type ATPase (EC 3.6.3.4), Cu(I)-responsive transcriptional regulator |
| 11 | 143186 | 149956 | *S*. Cubana, *S*. Typhimurium, *S*. Aantum, *S*. Enteritidis | 57.2 | 2-aminoethylphosphonate ABC transporter permease protein II (TC 3.A.1.9.1), 2-aminoethylphosphonate ABC transporter permease protein I (TC 3.A.1.9.1), 2-aminoethylphosphonate ABC transporter ATP-binding protein (TC 3.A.1.9.1), 2-aminoethylphosphonate ABC transporter periplasmic binding component (TC 3.A.1.9.1), 2-aminoethylphosphonate uptake and metabolism regulator, 2-aminoethylphosphonate:pyruvate aminotransferase (EC 2.6.1.37), Phosphonoacetaldehyde hydrolase (EC 3.11.1.1) |
| 11 | 238607 | 259753 | *S*. Tennessee, *S*. Weltevreden, *S*. Agona, *S*. Paratyphi B, *S*. Enteritidis | 51.7 | DNA-binding transcriptional activator of the allD operon, Ureidoglycolate hydrolase (EC 3.5.3.19), Negative regulator of allantoin and glyoxylate utilization operons, Glyoxylate carboligase (EC 4.1.1.47), Hydroxypyruvate isomerase (EC 5.3.1.22), 2-hydroxy-3-oxopropionate reductase (EC 1.1.1.60), Probable metabolite transport protein, Allantoin permease, Allantoinase (EC 3.5.2.5), xanthine/uracil permease family protein, Glycerate kinase (EC 2.7.1.31), Ureidoglycine aminohydrolase, Allantoate amidohydrolase (EC 3.5.3.9), Ureidoglycolate dehydrogenase (EC 1.1.1.154), hypothetical protein, Protein fdrA, FIG074102: hypothetical protein, FIG074102: hypothetical protein, Carbamate kinase (EC 2.7.2.2) |
| 11 | 264307 | 266103 | *S*. Anatum, *S*. Newport, *S*. Typhimurium, *S*. Agona, *S*. Enteritidis | 51.4 | Putative outer membrane protein, Putative inner membrane protein, Putative membrane-bound metal-dependent hydrolases |
| 11 (inserted adjacent to tRNA-Arg-TCT) | 273434 | 277236 | *S*. Typhimurium, *S*. Newport, *S*. Thompson, *S*. Agona, *S*. Heidleberg, *S*. Weltevreden, *S*. Paratyphi A, *S*. Enteritidis | 43.1 | Fimbriae-like periplasmic protein SfmF, Transcriptional regulator of fimbriae expression FimZ (LuxR/UhpA family), Transcriptional regulator of fimbriae expression FimY, Putative diguanylate cyclase/phosphodiesterase domain 0, Fimbriae W protein, FIG01046307: hypothetical protein |
| 15 | 1 | 11,176 (end) | *S*. Kentucky plasmid pCVM29188_146 | 50.2 | TnpA, Mobile element protein, Aminoglycoside 3'-phosphotransferase @ Streptomycin 3'-kinase StrA, Aminoglycoside 3'-phosphotransferase 2 @ Streptomycin 3'-kinase StrB, Replication regulatory protein repA2 (Protein copB), RepA1, Prevent host death protein, Phd antitoxin # D, Hypothetical protein, YacB, Colicin protein, Immunity protein |
| 40 | 1 | 6,953 (end) |  | 38.8 | Sodium/glutamate symport protein, hypothetical protein, hypothetical protein, FIG00731654: hypothetical protein, Transcriptional regulator, ArsR family, Tetracycline efflux protein TetA, Transcriptional regulator, TetR family, Right origin-binding protein |
| 6 | 1 | 55,340 (end) |  | 52.8 | Chromosome (plasmid) partitioning protein ParA, ParB, Putative antirestriction protein, Hypothetical plasmid protein, Single-stranded DNA-binding protein, Adenine-specific methyltransferase, PsiA protein,  X polypeptide, IncF plasmid conjugative transfer mating signal transduction protein TraM, IncF plasmid conjugative transfer regulator TraJ, TraY, IncF plasmid conjugative transfer pilin protein TraA, IncF plasmid conjugative transfer pilus assembly protein TraL, TraE, TraK, TraB, TraV, TraC, TraW, TraU, TraF, IncF plasmid conjugative transfer protein TraP, TrbD, TrbG, TraR, TrBI, TrbC, TraN, TrbE, TraQ, TrbB, TrbJ, TraG, TraD, Putative conjugative transfer protein TraH, IncF plasmid conjugative transfer surface exclusion protein TraS, IncF plasmid conjugative transfer surface exclusion protein TraT, IncF plasmid conjugative transfer DNA-nicking and unwinding protein TraI, IncF plasmid conjugative transfer pilin acetylase TraX, IncF plasmid conjugative transfer fertility inhibition protein FinO, IncF plasmid conjugative transfer fertility inhibition protein FinO, YihA, Moble element protein |
| 31 | 1 | 15,458 (end) |  | 51.2 | Manganese ABC transporter, periplasmic-binding protein SitA, Manganese ABC transporter, ATP-binding protein SitB, Manganese ABC transporter, inner membrane permease proteins SitC and SitD, Enolase,  Citrate:6-N-acetyl-6-N-hydroxy-L-lysine ligase, alpha subunit, aerobactin biosynthesis protein IucA @ Siderophore synthetase superfamily, group A @ Siderophore synthetase large component, acetyltransferase, N6-hydroxylysine O-acetyltransferase, aerobactin biosynthesis protein IucB @ Siderophore synthetase small component, acetyltransferase,  Citrate:6-N-acetyl-6-N-hydroxy-L-lysine ligase, alpha subunit, aerobactin biosynthesis protein IucA @ Siderophore synthetase superfamily, group C @ Siderophore synthetase component, ligase,  L-lysine 6-monooxygenase [NADPH], aerobactin biosynthesis protein IucD @ Siderophore biosynthesis protein, monooxygenase, Aerobactin siderophore receptor IutA @ TonB-dependent siderophore receptor, Mobile element protein |
| 30 | 1 | 49,445(end) |  | 46.8 | FIG01046320: hypothetical protein, YdeA protein, YdfA protein, PhnO protein, FIG01047927: hypothetical protein, hypothetical protein, NTD biosynthesis operon putative oxidoreductase NtdC (EC 1.-.-.-), Probable microcin H47 secretion ATP-binding protein, Putative secretion permease, hypothetical protein, YacB, FIG01046320: hypothetical protein, 2-keto-3-deoxy-D-arabino-heptulosonate-7-phosphate synthase I alpha (EC 2.5.1.54), Outer Membrane Siderophore Receptor IroN, Periplasmic esterase IroE, Trilactone hydrolase IroD, ABC transporter protein IroC, Glycosyltransferase IroB, hypothetical protein, hypothetical protein, Mobile element protein, Mobile element protein, hypothetical protein, Lipoprotein Bor, hypothetical protein, Mobile element protein, Mobile element protein, Putative metal chaperone, involved in Zn homeostasis, GTPase of COG0523 family, hypothetical protein, putative transposase, Putative cytoplasmic protein, RND efflux system, outer membrane lipoprotein CmeC, Macrolide export ATP-binding/permease protein MacB (EC 3.6.3.-), Macrolide-specific efflux protein MacA, hypothetical protein, Mobile element protein, Mobile element protein, Mobile element protein, COG2801: Transposase and inactivated derivatives, Protease VII (Omptin) precursor (EC 3.4.23.49), Protease VII (Omptin) precursor (EC 3.4.23.49), Nucleoside-diphosphate-sugar epimerases, Putative transcriptional regulator  Resolvase, RepFIB replication protein A, hypothetical protein, Mobile element protein, Error-prone, lesion bypass DNA polymerase V (UmuC) |
| 1 | 1 | 60,793 (end) | *S.* Kentucky plasmid CVM29188_101 | 50 | Incl1 plasmid conjugative transfer prepilin PilS, Incl1 plasmid conjugative transfer inner membrane protein PilR, Incl1 plasmid conjugative transfer ATPase PilQ, Incl1 plasmid pilus assembly protein PilP, PiIO, Incl1 plasmid conjugative transfer lipoprotein PilN, Incl1 plasmid conjugative transfer protein PilM, PilL, PilJ, PilI, PilK, TraC, TraA, Incl1 plasmid conjugative transfer NusG-type transcription antiterminator TraB, Replication initiation protein, Hypothetical protein, YacB,  YadA, Mobile element protein, Beta-lactamase, Outer membrane lipoprotein Blc, Quaternary ammonium compound-resistance protein SugE, 3',5'-cyclic-nucleotide phosphodiesterase, YdeA protein, YdfA protein, CcdA protein (antitoxin to CcdB), CcdB toxin protein, Resolvase, ybiA, Putative stability/partitioning protein encoded within prophage CP-933T, Stable plasmid inheritance protein, Error-prone, lesion bypass DNA polymerase V (UmuC), Error-prone repair protein UmuD, Adenine-specific methyltransferase, Putative cytoplasmic protein, YcgB, Putative antirestriction protein, Single-stranded DNA-binding protein, Putative plasmid stabilization protein, PsiB protein, PsiA protein, Transposase,  Antirestriction protein ArdA, YDFB protein, Z1226 protein, Nickel ABC transporter, periplasmic nickel-binding protein NikA , |
| 43 | 1 | 35,477 (end) |  | 49.7 | Hypothetical protein, TrbB, TrbA proteins, Mobile element protein, Phage minor tail protein, Surface exclusion protein, IncI1 plasmid conjugative transfer integral membrane protein TraY, IncI1 plasmid conjugative transfer protein TraX, TraW, TraV, TraU, TraT, TraS, TraR, TraQ, TraP, TraO, TraN, TraM and TraL, IncI1 plasmid conjugative transfer DNA primase,  Plasmid conjugative transfer endonuclease, IncI1 plasmid conjugative transfer protein TraJ, TraI, TraH, TraG, TraF and TraE, Shufflon-specific DNA recombinase |
| 23b | 1 | 24,098 (end) | *S.* Kentucky plasmid CVM29188_46 | 37.9 | Large repetitive protein, Predicted DNA-binding transcriptional regulator, IS1327 transposase, Protein gp49, Putative type-I secretion protein,  ABC Transporter, Replication regulatory protein repA2, RepA1, Methyl-accepting chemotaxis protein I (serine chemoreceptor protein) |
| 23 | 1 | 8,113 (end) |  | 46.5 | CcdA protein (antitoxin to CcdB), CcdB toxin protein, Permease of the drug/metabolite transporter (DMT) superfamily, Transposase and inactivated derivative |
| 30 | 1 | 4,233 (end) |  | 54.8 | Mobile element proteins, Hypothetical proteins, Mobilization protein mbeD, |
| 21 | 1 | 8,218 (end) |  | 45.6 | Hypothetical protein, Nicotinamidase family protein YcaA, Nickel ABC transporter, periplasmic nickel-binding protein NikA, PI protein, Plasmid partition protein ParG, Chromosome (plasmid) partitioning protein ParA |
